# Identifying Putative Causal Links between MicroRNAs and Severe COVID-19 Using Mendelian Randomization

**DOI:** 10.3390/cells10123504

**Published:** 2021-12-11

**Authors:** Chang Li, Aurora Wu, Kevin Song, Jeslyn Gao, Eric Huang, Yongsheng Bai, Xiaoming Liu

**Affiliations:** 1 USF Genomics & College of Public Health, University of South Florida, Tampa, FL 33612, USA; 2 Emma Willard School, Troy, NY 12180, USA; aurora.wuhaoshu@gmail.com; 3 Credit Suisse, New York, NY 10010, USA; kevinmsong@gmail.com; 4 Simsbury High School, Simsbury, CT 06070, USA; 23gaoj@gmail.com; 5 James E. Taylor High School, Katy, TX 77450, USA; shanqi15@yahoo.com; 6 Next-Gen Intelligent Science Training, Ann Arbor, MI 48105, USA; 7 Department of Biology, Eastern Michigan University, Ypsilanti, MI 48197, USA

**Keywords:** microRNA, SARS-CoV-2, COVID-19, biomarker, Mendelian randomization

## Abstract

The SARS-CoV-2 (COVID-19) pandemic has caused millions of deaths worldwide. Early risk assessment of COVID-19 cases can help direct early treatment measures that have been shown to improve the prognosis of severe cases. Currently, circulating miRNAs have not been evaluated as canonical COVID-19 biomarkers, and identifying biomarkers that have a causal relationship with COVID-19 is imperative. To bridge these gaps, we aim to examine the causal effects of miRNAs on COVID-19 severity in this study using two-sample Mendelian randomization approaches. Multiple studies with available GWAS summary statistics data were retrieved. Using circulating miRNA expression data as exposure, and severe COVID-19 cases as outcomes, we identified ten unique miRNAs that showed causality across three phenotype groups of COVID-19. Using expression data from an independent study, we validated and identified two high-confidence miRNAs, namely, hsa-miR-30a-3p and hsa-miR-139-5p, which have putative causal effects on developing cases of severe COVID-19. Using existing literature and publicly available databases, the potential causative roles of these miRNAs were investigated. This study provides a novel way of utilizing miRNA eQTL data to help us identify potential miRNA biomarkers to make better and early diagnoses and risk assessments of severe COVID-19 cases.

## 1. Introduction

COVID-19, a disease caused by the SARS-CoV-2 virus, has grown into a worldwide pandemic that has infected more than 200 million people and caused more than 4.4 million deaths since December 2019. Most infected cases of COVID-19 are asymptomatic or show mild symptoms, such as fever, cough, shortness of breath and muscle ache [1]. However, a small subset of the cases may develop more severe and critical disease courses, with dyspnea and hypoxemia as the most common symptoms [2,3]. Evidence has shown that early diagnosis and antiviral treatment of such cases can alleviate severe symptoms and improve prognosis [4,5]. While clinical assessments, including radiological features, are crucial to identifying suspicious cases, laboratory biomarkers can provide a more objective and sometimes more accessible means of identifying potential severe cases [6]. As COVID-19 is now classified as a multisystem disease, different biomarkers can be designed to measure various aspects of disease severity. For example, the most common and easily accessible biomarkers for COVID-19 are from blood samples, such as hemoglobin and lymphocyte counts [7]. Biomarkers based on these signatures, such as a high neutrophil-to-lymphocyte ratio, can be suggestive of disease severity. Additionally, many protein-based biomarkers, such as C-reactive protein (CRP) and cytokines targeting inflammatory factors, have been explored [8]. While these biomarkers have their own advantages, a crucial type of biomarker, microRNAs, have been overlooked despite their multiple clinical advantages over other biomarkers [9]. First, they are easily sourced from bodily fluids such as blood and urine. Second, they show high specificity as different miRNAs usually target various genes involved in diverse biological pathways. Third, the technologies for detecting nucleic acids already exist, e.g., RNA-sequencing to quantify miRNA expression levels, compared to new antibodies usually required for protein-based biomarkers.

MicroRNAs (miRNA) are a class of short non-coding RNA with an average length of approximately 22 nucleotides. MiRNAs can selectively bind to the 3′ untranslated regions (UTRs) of protein-coding genes to post-transcriptionally repress their expression [10]. Currently, more than 2000 mature miRNAs have been reported and curated at miRbase for humans [11]. MiRNAs have shown a broad spectrum of functions and have been implicated in numerous diseases from cancers to viral infections [12,13,14,15]. Additionally, miRNAs have been widely investigated for their potential role as biomarkers in other diseases [9,16,17]. The most commonly studied clinical source for miRNA is blood (circulating miRNAs). These circulating miRNAs are present in human plasma and serum in a highly stable form. They are resistant to degradation, making miRNAs an ideal type of noninvasive biomarker [18]. Moreover, recent studies have explored the association between miRNAs and COVID-19 infection. Multiple miRNAs were reported to be differentially expressed in COVID-19 patients using peripheral blood samples [19]. Potential antiviral therapeutic roles of miRNAs have also been explored [20]. This evidence suggests that miRNAs as key regulators of human genes could be a valuable target to help us better understand the mechanisms of COVID-19 development and work as specific biomarkers for COVID-19 severity and patient risk classification.

**Figure 1:**
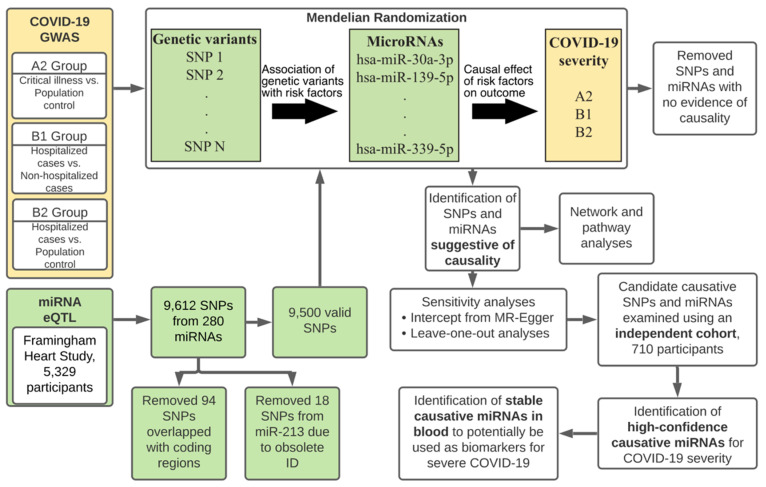
Flowchart of the study design. Green boxes show the processes related to risk factor (miRNAs) and yellow boxes show the processes related to outcome (COVID-19).

In this study, to investigate the potential of miRNAs in predicting severe COVID-19 cases, we adopted a two-sample Mendelian randomization (MR) approach to explore the causal relationship between circulating miRNAs and COVID-19 severity in the presence of potential confounding factors (Figure 1) [21]. Using summary statistics from genome-wide association studies (GWAS), we identified multiple putative causal miRNAs that can contribute to the severity of COVID-19. We further validated our findings using an independent GWAS. To our knowledge, this is the first paper to investigate the causal relationship between miRNAs and COVID-19.

## 2. Materials and Methods

### 2.1. MiRNA eQTL Data Retrieval

The miRNA expression quantitative loci (eQTLs) are genome-wide analyses of variants associated with the expression level of miRNAs. We retrieved the miRNA eQTL data from a previous population-based study [22], the Framingham Heart Study (FHS). Briefly, there were 5329 participants, and their whole blood samples were analyzed using quantitative reverse transcription PCR (qRT-PCR) to quantify miRNA expression levels. A total of ~10 million single nucleotide polymorphisms (SNPs) passed quality control processes for each of the 280 high-quality miRNAs. A Benjamini–Hochberg corrected false discovery rate (FDR) of 0.1, which corresponds to a raw *p*-value of 6.6 × 10^−5^, was used as a cutoff to minimize false-positive discoveries. Since the raw association data were unavailable, only these significant SNPs were retrieved from the publication [22]. This study only focused on cis-miRNA-eQTLs, defined as SNPs located within 1 megabase (MB) on either side of the corresponding miRNA. The reason for us to focus on cis-eQTL is to minimize the potential horizontal pleiotropic effect of the candidate SNPs. SNPs located in coding regions with consequences of synonymous or missense were removed to further reduce the potential pleiotropic effects. This data set is referred to as the original data.

Additionally, to validate our findings, we retrieved another independent miRNA eQTL data set from a recent study [23]. The data were requested directly from the authors which included the complete eQTL results for each miRNA analyzed. This study comprehensively examined 2083 mature human miRNAs and their expression levels in blood samples among 710 unrelated people of European ancestry. Even though this study is more recent and covered more miRNAs, we still chose the FHS data (original data) as our primary study population given its larger sample size (*n* = 5329 vs. *n* = 710) and greater power in detecting the relationship between miRNAs and COVID-19. This data set is referred to as the validation data.

Since the miRNA IDs used in our validation data differed from those used in the original data, we converted miRNA IDs between the two datasets using miRCarta v1.1 [24].

### 2.2. COVID-19 GWAS Data Retrieval

GWAS summary statistics for COVID-19 cases were retrieved from the COVID-19 Host Genetics Initiative release 6 (https://www.covid19hg.org/results/r6/COVID-19 (accessed on 18 October 2021)). These GWAS data have already been meta-analyzed so that results from different study centers were combined to facilitate subsequent analyses and ensure the confidentiality of personal data. The meta-analysis results were obtained for three phenotype groups as defined by the original paper [25], namely the A2, B1 and B2 groups (Table 1). Even though the C2 group had the largest sample size, it used reported COVID-19 as cases. Therefore, the C2 group was removed from further analyses to ensure the high-quality of the analyses. The A2 group corresponds to GWAS analysis using critically ill COVID-19 patients as cases and the general population as controls. The B1 group corresponds to GWAS analysis using hospitalized COVID-19 patients as cases and non-hospitalized COVID-19 as controls. The B2 group corresponds to GWAS analysis using hospitalized COVID-19 patients as cases and the general population as controls. Briefly, critical illness was defined as hospitalized cases with confirmed SARS-CoV-2 infection requiring respiratory support or whose death was associated with COVID-19. The hospitalized group was defined as hospitalized patients with confirmed SARS-CoV-2 infection. The non-hospitalized COVID-19 group was defined as having a reported SARS-CoV-2 infection without clinical confirmation. The population control was defined as those with unknown SARS-CoV-2 status or infection-negative people from questionnaires or electronic health records. All SNPs that passed the quality filters described in their corresponding papers were included in subsequent analyses in our study.

### 2.3. Mendelian Randomization

To examine the influence of miRNA expression levels on the risk of COVID-19, we adopted Mendelian randomization (MR). MR uses genetic variants as instrumental variables (IVs) to uncover the causal relationship between an exposure and an outcome [26]. In MR studies, IVs are usually SNPs that are associated with the exposure (miRNA) and observed in outcome (COVID-19) GWAS. Using IVs as surrogates of the exposure can minimize the confounding effects associated with the exposure and outcome, which enables unbiased estimation of causality. A one-sample MR was commonly applied to studies where the exposure, outcome, and genetic variants were measured in the same samples. However, it is often difficult and expansive to obtain such data to conduct the analysis. Therefore, a more versatile approach, two-sample MR, was developed to perform MR on exposures and outcomes measured in different individuals. One major advantage of a two-sample MR is that it can use summary statistics from GWAS data to make causal inferences, which takes advantage of the proliferation of publicly available GWAS data.

Using previously curated summary statistics from different GWAS data mentioned previously, we leveraged two MR methods to study the relationship between miRNA and COVID-19, namely inverse variance weighted (IVW) MR [27] and MR-Egger regression [28]. IVW is the traditional MR method that combines the Wald ratio estimates using a meta-analysis approach. The final estimate of the IVW MR is a weighted sum of the contribution of each individual IV (SNP). The IVW method requires all IVs to satisfy these three assumptions, namely, the relevance assumption (IVs are associated with the risk factors), independence assumption (no confounders between IVs and the outcome) and exclusion restriction (no horizontal pleiotropic effects). While the independence assumption can be difficult to evaluate, the MR-Egger regression is a more robust method in the presence of pleiotropic effects with a sacrifice in statistical power. MiRNA eQTLs for 280 miRNAs that passed the genome-wide significance level were used as IVs. To identify independent IVs, we performed linkage disequilibrium (LD) clumping using PLINK 1.9 [29], and independent SNPs that are strong predictors for miRNA expression levels in blood samples were obtained to satisfy the relevance assumption. We used r^2^ < 0.5 and a 10 kb clumping window and the European reference panel from the 1000 Genomes Project to discard variants in LD with another variant while keeping the SNP with the smallest *p*-value in each window. The same set of SNPs that are associated with miRNAs was retrieved from the COVID-19 GWAS data. Then the exposure and outcome data were harmonized to ensure common effect alleles. Palindromic SNPs with MAF > 0.42 were removed due to ambiguity in inferring the strand identity. We claim there is putative causation between miRNA and COVID-19 if all following criteria are met: (1) Significant miRNA and COVID-19 association to have *p*-value less than 0.05/75 = 0.00067 for IVW test. (2) Estimated effect size for both models (IVW and MR-Egger) to have the same direction of association. (3) At least 3 SNPs must be present for MR tests. (4) High-confidence relationship was claimed only if validation study showed significant results. These stringent criteria are expected to minimize the effect of potential violation of the assumptions mentioned previously. All MR analyses were performed using R package TwoSampleMR v.0.5.6 available at https://mrcieu.github.io/TwoSampleMR/ (accessed on 29 October 2021) [30].

### 2.4. Network and Pathway Analyses

To study the functional impact of the identified putatively causal miRNAs, we first identified their target genes using miRNet 2.0 and its web-service [31], available at https://www.mirnet.ca/miRNet/home.xhtml (accessed on 24 November 2021). Only experimentally validated target genes were considered as represented by the miRTarBase v8.0 database [32]. Next, a miRNA-gene network was constructed using the same web service. To ensure that only the most important genes were included, degree centrality and betweenness were set to greater than or equal to 1. We further performed functional enrichment analysis using the Gene Ontology (GO) Biological Process (BP) pathways using the identified target genes. To control for multiple comparisons, a false discovery rate (FDR) <0.05 was used.

### 2.5. Sensitivity Analyses

We performed a series of sensitivity analyses to examine whether the observed putative causal relationship is prone to disturbance using R package TwoSampleMR [30]. All significant miRNAs identified using the original data were analyzed. First, an intercept test from MR-Egger regression was adopted to check for potential horizontal pleiotropy. Second, a leave-one-out MR was performed to check if a single SNP is driving the observed causal effects. For each miRNA, all its eQTLs used in the initial analysis were removed one by one, and MR analysis was performed using the rest of the SNPs. All the new MR estimates were compared with the initial MR estimates which used all SNPs to determine if a single SNP was dominant in the observed association. If all the new MR estimates did not change substantially from the initial analysis, we can conclude that our results are robust to disturbance. Additionally, the validation data were used to identify high-confidence causal miRNAs. The same clumping and harmonization steps were performed (see Section 2.3).

## 3. Results

### 3.1. Description of GWAS SNPs

A total of 9612 SNPs were collected as instrumental variables (IVs) from the original data (miRNA eQTL data). SNPs located in coding regions, either synonymous or missense SNPs were removed to minimize their potential horizontal pleiotropic effects. There were 9518 SNPs located in regions without direct association with coding genes. SNPs associated with miR-213 were removed due to failure to map the miRNA to a valid miRNA ID. In total, there were 9500 SNPs remained from miRNA eQTL data and were used in subsequent analyses (Figure 1). These 9500 SNPs were significantly associated with the expression of 75 circulating human mature miRNAs which came from 71 unique miRNA families. The miR-99-5p/100-5p, miR-125-5p, miR-130-3p/301-3p/454-3p and miR-148-3p/152-3p families have two mature miRNAs, while all other miRNA families have only one mature miRNA included in the analysis. Additionally, different numbers of eQTL loci for each mature miRNA were reported, with hsa-miR-1270 having the most SNPs (*n* = 756) while hsa-miR-323a-3p and hsa-miR-337-3p have the least number of SNPs (*n* = 1), which were removed in subsequent analyses.

### 3.2. MiRNAs Can Covey Both Protective and Harmful Effects in COVID-19 Severity

**Figure 2:**
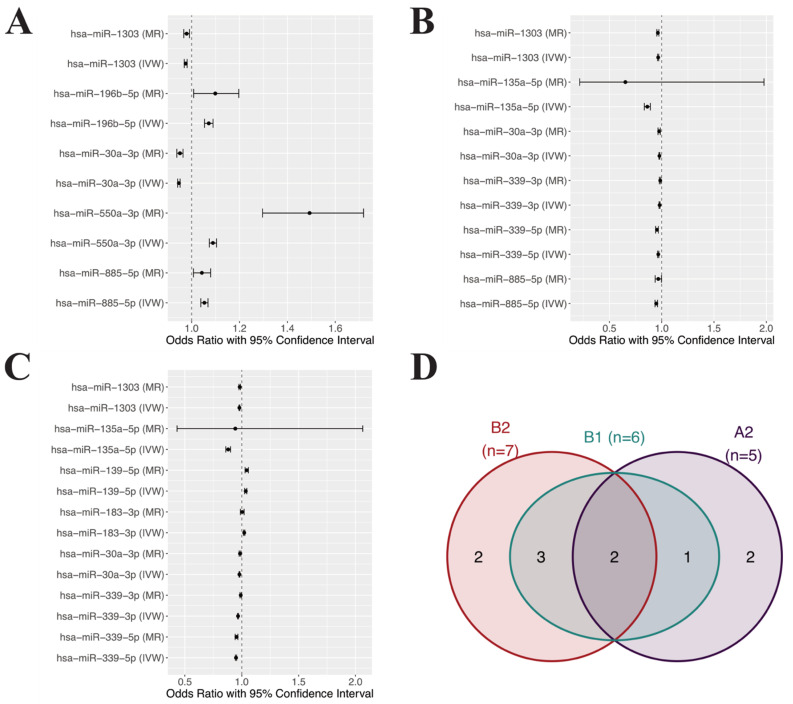
Protective and harmful effects of miRNAs on COVID-19 severity. The circle indicates the point estimate of OR and the whisker shows its 95% confidence interval. (**A**) Significant causal relationships identified in the A2 phenotype group. (**B**) Significant causal relationships identified in B1 phenotype group. (**C**) Significant causal relationships identified in B2 phenotype group. (**D**) Overlapping putatively causal miRNAs between the three phenotype groups. Numbers inside the circles represent the number of overlapping or unique miRNAs. MR: MR-Egger, IVW: Inverse variance weighted MR.

Using IVW and MR-Egger regression, we listed all significantly associated miRNAs in Figure 2A using the A2 phenotype group (critical illness vs. population control). After Bonferroni multiple testing correction, five blood miRNA markers showed significant associations with an elevated risk of critical illness of COVID-19 (Table S1). The strongest effect size was observed for hsa-miR-550a-3p where a one standard deviation (SD) increase was associated with approximately 50% higher odds of severe COVID-19. Similar effects were observed for hsa-miR-196b-5p and hsa-miR-885-5p. In contrast, a 1 SD increase in hsa-miR-30a-3p was associated with 5% decreased odds of severe COVID-19. A similar protective effect was observed for hsa-miR-1303.

Following the same analysis pipeline, in the B1 phenotype group (hospitalized COVID-19 vs. non-hospitalized reported COVID-19), six circulating miRNAs were identified, and both MR-Egger regression and IVW methods showed the same direction of associations (Figure 2B). Interestingly, all miRNAs observed in this analysis had a protective effect against severe COVID-19. With hsa-miR-135a-5p had the lowest OR (0.862) indicating that with a 1 SD increase in the miRNA, the odds of hospitalization decreased by 14%. With only three IVs, predictions made by MR-Egger method for hsa-miR-135a-5p showed an extremely wide confidence interval. However, the point estimation, which is the most confident single estimation, showed the most potent protective effect (OR = 0.652). All other miRNAs seemed to have similar protective effects with approximately 2–5.2% decreased odds of hospitalization with a 1 SD increase in miRNA expressions.

We identified seven putative causal relationships using the B2 phenotype group (hospitalized COVID-19 vs. population control, Figure 2C). Among the seven miRNAs, 5 showed a protective effect with 2–12% decreased odds of hospitalization compared to healthy population control with a 1 SD increase in hsa-miR-1303, hsa-miR-135a-5p, hsa-miR-30a-3p, hsa-miR-339-3p, and hsa-miR-339-5p. Conversely, two miRNAs were associated with 2.2–3.5% increased odds of hospitalization, including hsa-miR-139-5p and hsa-miR-183-3p. A total of 10 unique miRNAs were observed from the previous analyses and they will be used as candidates of potential causative miRNAs for COVID-19 severity in subsequent analyses (Figure 2D).

### 3.3. Viral Infection Related Pathways Were Significantly Enriched in Network Analysis

**Figure 3:**
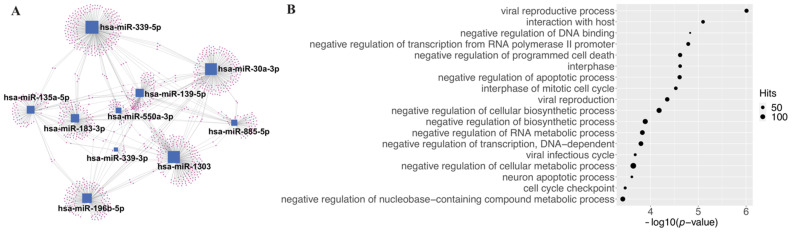
Results of pathway and network analyses for the 10 candidate miRNAs. (**A**) MiRNA-gene network for ten potentially causal miRNAs of severe COVID-19. Blue squares indicate miRNAs, and red dots indicate corresponding target genes. Edges between each blue square and red dot represent that the gene can be targeted by the miRNA. (**B**) Function enrichment analysis of target genes on the biological process (BP) GO pathways (only the top 18 enriched pathways are shown).

We next identified potential target genes for the ten candidate causal miRNAs, and miRNet was adopted to construct a miRNA-target gene network (Figure 3A) [31]. A total of 1130 unique genes were identified to be regulated by these ten miRNAs. MiRNAs that can target the most genes are hsa-miR-339-5p with 235 experimentally validated target genes, followed by hsa-miR-1303 which can target 201 genes, and hsa-miR-30a-3p which can target 194 genes. To further elucidate the function of all these identified potentially causal miRNAs and their target genes, functional enrichment analysis of related Gene Ontology (GO) terms for these genes was performed. The GO terms are standardized terms to describe known functions of genes. These terms are commonly used in gene set enrichment analysis to identify biological pathways regulated by the gene set. The results show that genes regulated by these miRNAs are widely involved in multiple viral-infection-related pathways, such as “viral reproductive process”, “interaction with host”, “viral reproduction” and “viral infectious cycle” (Figure 3B), which indicated that miRNAs identified using the MR methods can capture meaningful biological information.

### 3.4. Two High-Confidence miRNAs Were Validated Using an Independent Cohort

Next, we validated our predictions by examining high-confidence miRNA-COVID-19 relationships that were observed in an independent study [23]. First, we collected genome-wide miRNA eQTL data for all ten candidate causal miRNAs. One miRNA, hsa-miR-550a-3p, was not covered in the validation study, and another miRNA, hsa-miR-339-3p, was identified to have a potential pleiotropic effect, and both were removed from subsequent analyses. This left us with eight candidate miRNAs for further validation. Second, we set 1 × 10^−5^ as the *p*-value cutoff to select relevant IVs. The same quality control processes were performed as we did in our original study. After data preprocessing, two out of the eight miRNAs were validated in this new study (Table 2 and Table S3). The first miRNA, hsa-miR-30a-3p showed a protective effect against severe COVID-19 with an OR equal to 0.84 (95% CI: 0.79–0.9), which was in accordance with results observed using original data (OR = 0.95, 95% CI: 0.94–0.96). The second miRNA, hsa-miR-139-5p, acted as a risk factor for severe COVID-19 with an OR equal to 1.1 (95% CI: 1.07, 1.13), which was also in accordance with results observed using original data (OR = 1.035, 95% CI: 1.026, 1.044).

## 4. Discussion

In this study, by combining GWAS datasets of COVID-19 severity and miRNA eQTL datasets with MR methods, we examined the potential causal roles of miRNAs in COVID-19 severity. We proposed our results by different phenotype groups of COVID-19, namely A2, B1 and B2. As expected, miRNAs identified using these three phenotype groups showed many overlaps (Figure 2D). Interestingly, with a total of 10 unique miRNAs, miRNAs identified using the B1 group (hospitalized vs. non-hospitalized COVID-19 cases, *n* = 6) completely overlapped with the other two comparisons with more extreme phenotypes, namely A2 (*n* = 5) and B2 (*n* = 7). This pattern of identified miRNAs can provide a means of validation for the data quality and show that the candidate miRNAs can likely reflect the actual disease severity. With this assumption, the two miRNAs exclusively in the B2 group (hsa-miR-139-5p and hsa-miR-183-3p) are more likely associated with only hospitalization risks, whereas the two miRNAs exclusively in the A2 group (hsa-miR-196b-5p and hsa-miR-550a-3p) are more likely related to the risk of critical illness. Additionally, two miRNAs showed significance in all three phenotype groups, namely hsa-miR-30a-3p and hsa-miR-1303, and their direction of association with severe COVID-19 was the same (protective).

**Figure 4:**
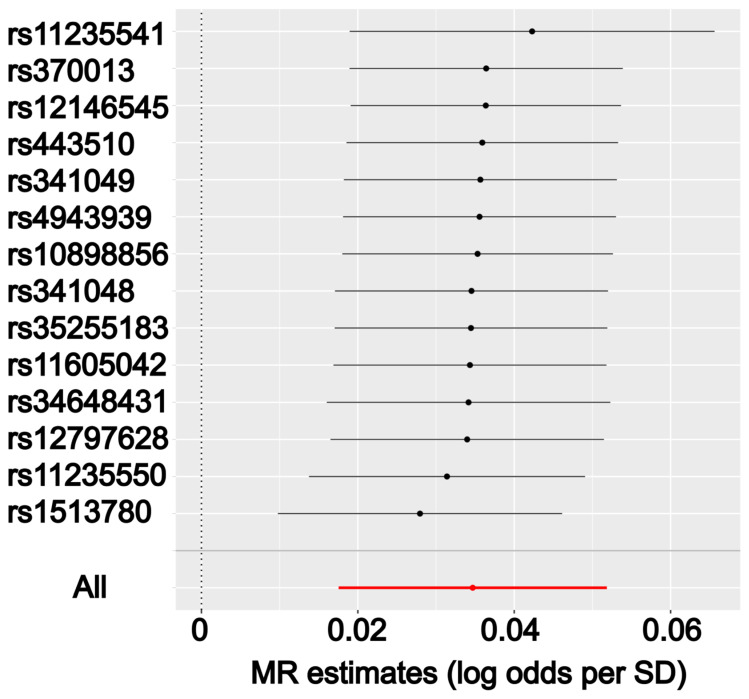
Results from leave-one-out sensitivity analysis. The y-axis shows the ID of the SNP to be excluded from analysis and the x-axis shows the MR estimates (odds ratios). The red line indicates the MR estimate using all SNPs. Hsa-miR-139-5p putatively leads to increased odds of hospitalization. Only one representative relationship was shown here. Complete results are available from the Supplementary Figures.

Mendelian randomization is prone to disturbance, and severe violation of the assumptions for IVs can significantly bias the results. To minimize such biases, we performed a series of informal and formal measures and analyses. First, we excluded SNPs in our analysis that were potentially associated with coding sequences, which can reduce the potential horizontal pleiotropic effects resulting from alternative causal pathways [33]. Second, SNPs in high LD with each other were removed, and only SNPs that showed the strongest association with the risk factors (miRNAs) were retained. This measure ensured the relevance of selected IVs to the risk factors. Third, we examined the shared targetome between the 10 candidate miRNAs and found that the degree of nodes for target genes (red dots) was low, with a maximum degree of 3 (Figure 2A). These sparse connections imply that the causal pathways established by each of these miRNAs are likely independent. In other words, the causal relationships between each miRNA and COVID-19 severity are likely to be independent, which indicates the validity of our MR method since such independence can result in minimized horizontal pleiotropic effects of IVs. Next, we formally tested the presence of potential horizontal pleiotropic effect of the IVs. We used the intercept from MR-Egger analysis as a measure of pleiotropic effect to further examine this assumption of the IVs [34]. An intercept of 0 shows no significant horizontal pleiotropy. As shown in Table S2, only 2 SNPs were detected as potentially having invalid IVs, namely hsa-miR-550a-3p in the A2 phenotype group analysis and hsa-miR-339-3p in the B2 phenotype group analysis. All other reported relationships were likely free from invalid IVs. Finally, we evaluated whether a single SNP drove the observed putative causal effects. Leave-one-out analyses were performed by removing one variant from each miRNA-phenotype group analysis and the causal effect was re-estimated. We found that all causal effects reported in this study showed robustness to disturbance that removing any single SNP did not significantly change the results, even for analyses with only 3 IVs (Figure 4, Supplementary Figures).

Using validation data, we identified two high confidence miRNAs, namely hsa-miR-30a-3p and hsa-miR-139-5p, which were causally associated with the severity of COVID-19. For the first miRNA, hsa-miR-30a-3p, while there was no reported direct link between the miRNA and COVID-19, experimental studies showed evidence that the miRNA can negatively regulate BAFF (B cell activating factor) by directly binding to the 3′UTR of the target gene [35]. BAFF is a key member of the TNF superfamily, which is involved in the activation and differentiation of B cells. It has been widely studied previously to be associated with autoimmunity. A recent publication showed that BAFF is also upregulated in severe COVID-19 cases [36]. This agrees with our observation that downregulation of hsa-miR-30a-3p can result in an upregulated BAFF level associated with severe COVID-19 cases. Interestingly, the hsa-miR-30a-3p was observed across all three phenotype groups compared in our original data, indicating that it is involved in a canonical pathway to be activated for severe COVID-19 cases. Similarly, while no direct association was reported between COVID-19 and hsa-miR-139-5p, a recent study reported two upregulated miRNAs in SARS-CoV-2 infected cells, and one of them was hsa-miR-139-5p [37]. This observation supports our MR analysis that the expression level of hsa-miR-139-5p is positively associated with COVID-19 severity.

**Figure 5:**
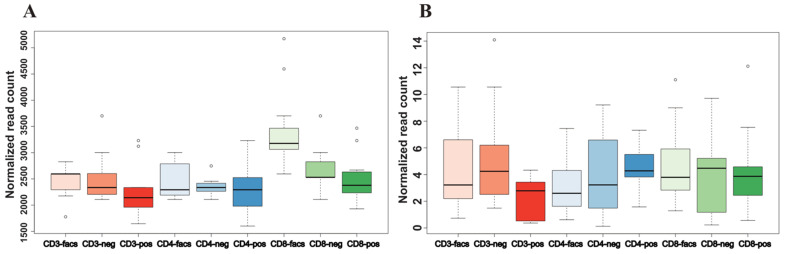
The normalized expression levels for two high-confidence miRNAs across different extraction techniques by PBMCs. (**A**) Hsa-miR-30a-3p. (**B**) Hsa-miR-139-5p. FACS: fluorescent activated cell sorting, neg: negative immunomagnetic selection, pos: positive immunomagnetic selection.

As MR analysis can imply causality between an exposure and an outcome, next we explored the possibility of using these two high-confidence miRNAs as biomarkers for COVID-19 severity. For whole blood/serum samples, both high-confidence miRNAs have been explored as potential biomarkers for different health conditions [38,39]. For RNA-sequencing profiling in peripheral blood mononuclear cells (PBMCs), the potential of these two miRNAs as biomarkers has not been investigated. Using the CellTypeAtlas web service (https://ccb-web.cs.uni-saarland.de/cf/ (accessed on 10 November 2021)), we found that both hsa-miR-30a-3p and hsa-miR-139-5p are highly expressed in various PBMCs, such as CD3, CD4, and CD8 cells [40]. The web service also provided the impact of different extraction techniques, including fluorescent activated cell sorting (FACS) and positive and negative immunomagnetic selection, on the variability of miRNA expression across these PBMCs. As shown in Figure 5, both miRNAs were stably expressed regardless of cell type and extraction technique, as indicated by the stable distributions of their expression levels across these different conditions. Given the accessibility of blood samples and the stability of these miRNAs under different experimental conditions in PBMCs, these two high-confidence miRNAs have the potential to serve as biomarkers for COVID-19 severity.

## 5. Conclusions

In this study, using the Mendelian randomization (MR) approach, we explored the causal link of circulating miRNAs on COVID-19 severity. By analyzing three phenotype groups with different COVID-19 severities, we identified ten mature miRNAs causally associated with COVID-19 severity and hospitalization. Our functional enrichment analysis identified multiple viral-infection-related pathways to be regulated by these miRNAs. We performed MR analyses in an independent cohort to validate our findings, and two high-confidence miRNAs, namely hsa-miR-30a-3p and hsa-miR-139-5p, were validated. Additional lines of evidence have confirmed the relevance of these miRNAs in the pathogenesis of COVID-19 severity. In accordance with previous evidence, hsa-miR-30a-3p is observed to have protective effects against severe COVID-19, whereas hsa-miR-139-5p can act as a risk factor for severe COVID-19. Last, we showed that these miRNAs are stable and have sufficient expression levels in multiple types of blood cells and therefore could potentially be used as clinical biomarkers for COVID-19 severity. Moreover, the analytical framework proposed in this study could assist in understanding COVID-19 severity and the discovery of novel biomarkers for clinical risk assessment and classification of COVID-19 patients.

## Figures and Tables

**Table 1:** Phenotype groups available for COVID-19 GWAS data.

Phenotype Groups	No. of Cases	No. of Controls	Case Group	Control Group
A2	8779	1,001,875	Critical illness	Population
B1	14,480	73,191	Hospitalized	Non-hospitalized reported COVID-19
B2	24,274	2,061,529	Hospitalized	Population
C2	112,612	2,474,079	Reported COVID-19	Population

**Table 2:** High-confidence set of miRNA biomarkers for severe COVID-19 identified using independent miRNA eQTL data.

miRNA	Phenotype Group	nSNPs *	Beta †	Se	*p*-Value	OR (95% CI) §
hsa-miR-30a-3p	A2	8	−0.174499	0.066973	0.009173	0.84 (0.79, 0.90)
hsa-miR-139-5p	B2	29	0.095454	0.025018	0.000136	1.10 (1.07, 1.13)

* The number of eQTLs used in the MR analysis for that miRNA. † A Beta/regression coefficient in the MR analysis. A positive value indicates that the miRNA shows a protective effect against severe COVID-19; a negative value indicates that the miRNA is a risk factor for severe COVID-19. § The odds ratio and its 95% confidence interval. When this confidence interval does not include 1, we claim the effect of miRNA on COVID-19 severity to be statistically significant.

## Data Availability

All data from the Framingham Heart Study were obtained from supplementary files within the published paper [22]; COVID-19 related GWAS data were obtained from the COVID-19 Host Genetics Initiative (https://www.covid19hg.org (accessed on 18 October 2021)); validation data were obtained from study [23] through request to the authors. All significant miRNAs and their statistics are available in the Supplementary Material associated with this paper.

## Supplementary Materials

The following are available online at https://www.mdpi.com/article/10.3390/cells10123504/s1, Table S1: Significant miRNAs associated with COVID-19 severity across all phenotype groups, Table S2: Intercept test results using MR-Egger regression for all significant miRNAs, Table S3: Validation results for all significant miRNAs. Supplementary Figures: Leave-one-out Mendelian randomization for all candidate miRNAs before quality assessment by phenotype groups (26 figures).
