# Supplementary material for: Identifying Putative Causal Links between MicroRNAs and Severe COVID-19 Using Mendelian Randomization
Source: Cells. 2021 Dec 11;10(12):3504. doi: 10.3390/cells10123504 (PMC8700362; doi:10.3390/cells10123504)
Supplement: Supplementary file 1 [file cells-10-03504-s001.zip › Supplementary Figures.zip!/A2_LOO/hsa-miR-1303A2.pdf]

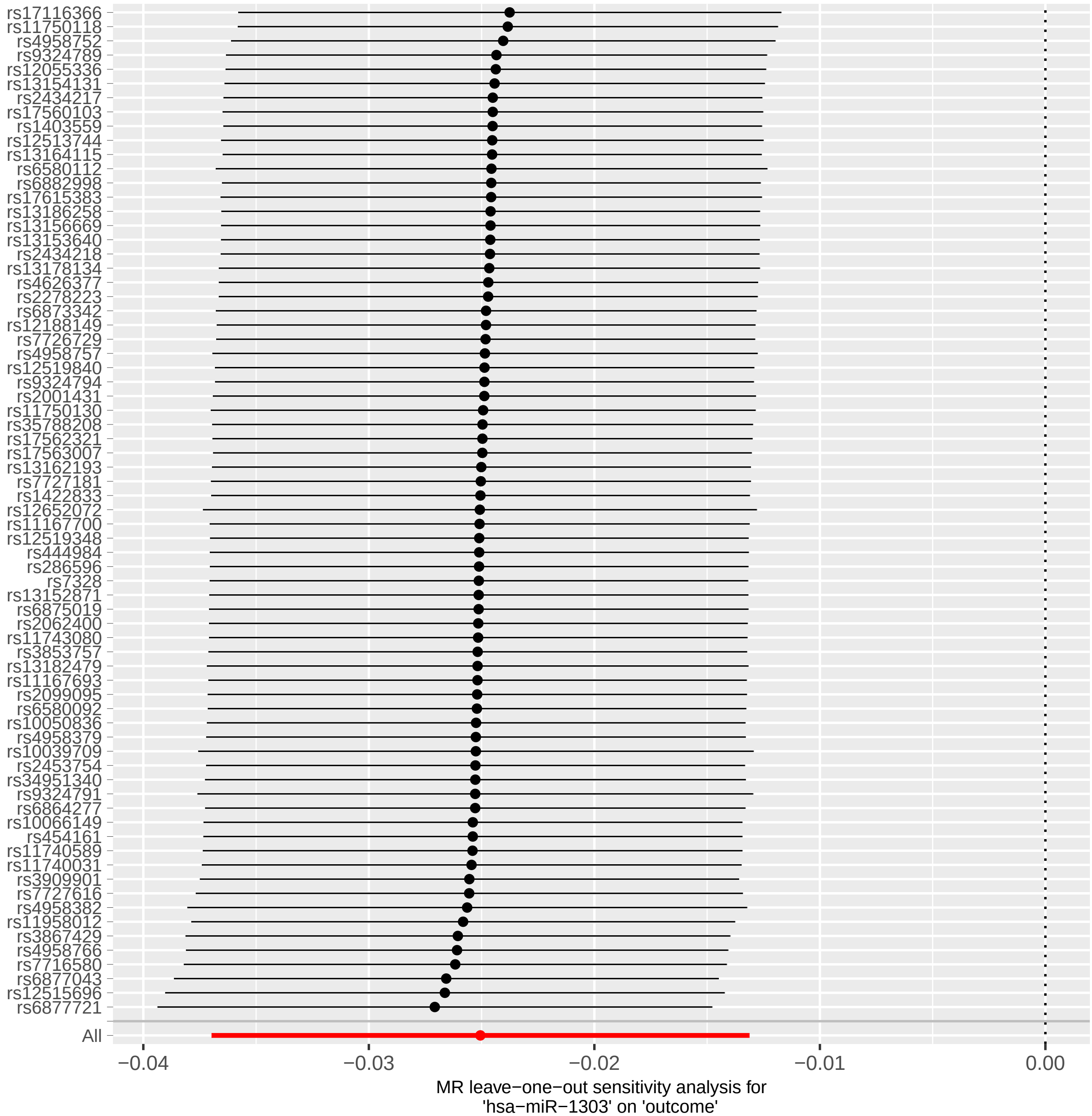
rs17116366
rs11750118
rs4958752
rs9324789
rs12055336
rs13154131
rs2434217
rs17560103
rs1403559
rs12513744
rs13164115
rs6580112
rs6882998
rs17615383
rs13186258
rs13156669
rs13153640
rs2434218
rs13178134
rs4626377
rs2278223
rs6873342
rs12188149
rs7726729
rs4958757
rs12519840
rs9324794
rs2001431
rs11750130
rs35788208
rs17562321
rs17563007
rs13162193
rs7727181
rs1422833
rs12652072
rs11167700
rs12519348
rs444984
rs286596
rs7328
rs13152871
rs6875019
rs2062400
rs11743080
rs3853757
rs13182479
rs11167693
rs2099095
rs6580092
rs10050836
rs4958379
rs10039709
rs2453754
rs34951340
rs9324791
rs6864277
rs10066149
rs454161
rs11740589
rs11740031
rs3909901
rs7727616
rs4958382
rs11958012
rs3867429
rs4958766
rs7716580
rs6877043
rs12515696
rs6877721
All
−0.04
−0.03
−0.02
−0.01
0.00
MR leave−one−out sensitivity analysis for
'hsa−miR−1303' on 'outcome'
