# Supplementary material for: Identifying Putative Causal Links between MicroRNAs and Severe COVID-19 Using Mendelian Randomization
Source: Cells. 2021 Dec 11;10(12):3504. doi: 10.3390/cells10123504 (PMC8700362; doi:10.3390/cells10123504)
Supplement: Supplementary file 1 [file cells-10-03504-s001.zip › Supplementary Figures.zip!/A2_LOO/hsa-miR-130a-3pA2.pdf]

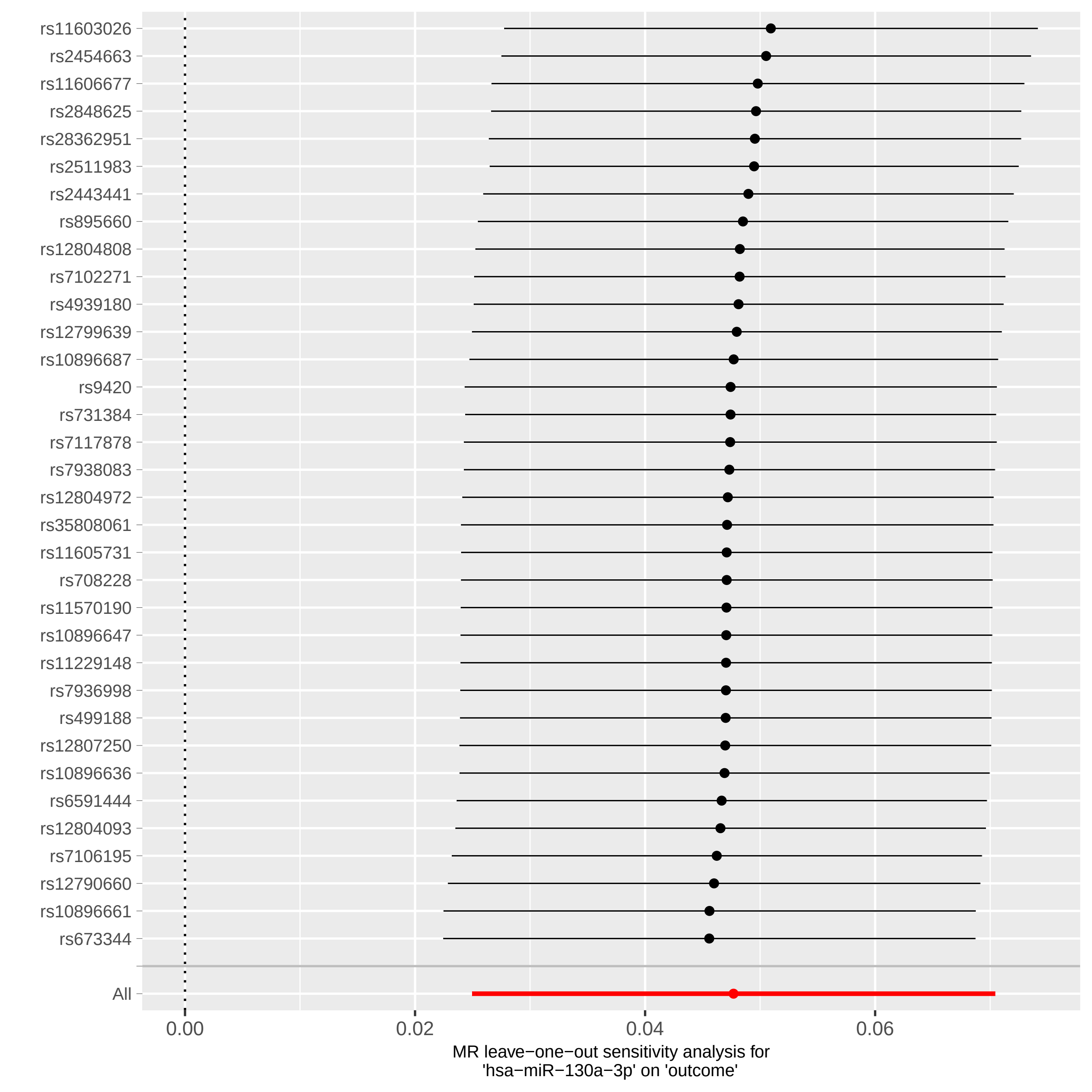
rs11603026
rs2454663
rs11606677
rs2848625
rs28362951
rs2511983
rs2443441
rs895660
rs12804808
rs7102271
rs4939180
rs12799639
rs10896687
rs9420
rs731384
rs7117878
rs7938083
rs12804972
rs35808061
rs11605731
rs708228
rs11570190
rs10896647
rs11229148
rs7936998
rs499188
rs12807250
rs10896636
rs6591444
rs12804093
rs7106195
rs12790660
rs10896661
rs673344
All
0.00
0.02
0.04
0.06
MR leave−one−out sensitivity analysis for
'hsa−miR−130a−3p' on 'outcome'
