# Supplementary material for: Identifying Putative Causal Links between MicroRNAs and Severe COVID-19 Using Mendelian Randomization
Source: Cells. 2021 Dec 11;10(12):3504. doi: 10.3390/cells10123504 (PMC8700362; doi:10.3390/cells10123504)
Supplement: Supplementary file 1 [file cells-10-03504-s001.zip › Supplementary Figures.zip!/A2_LOO/hsa-miR-148a-3pA2.pdf]

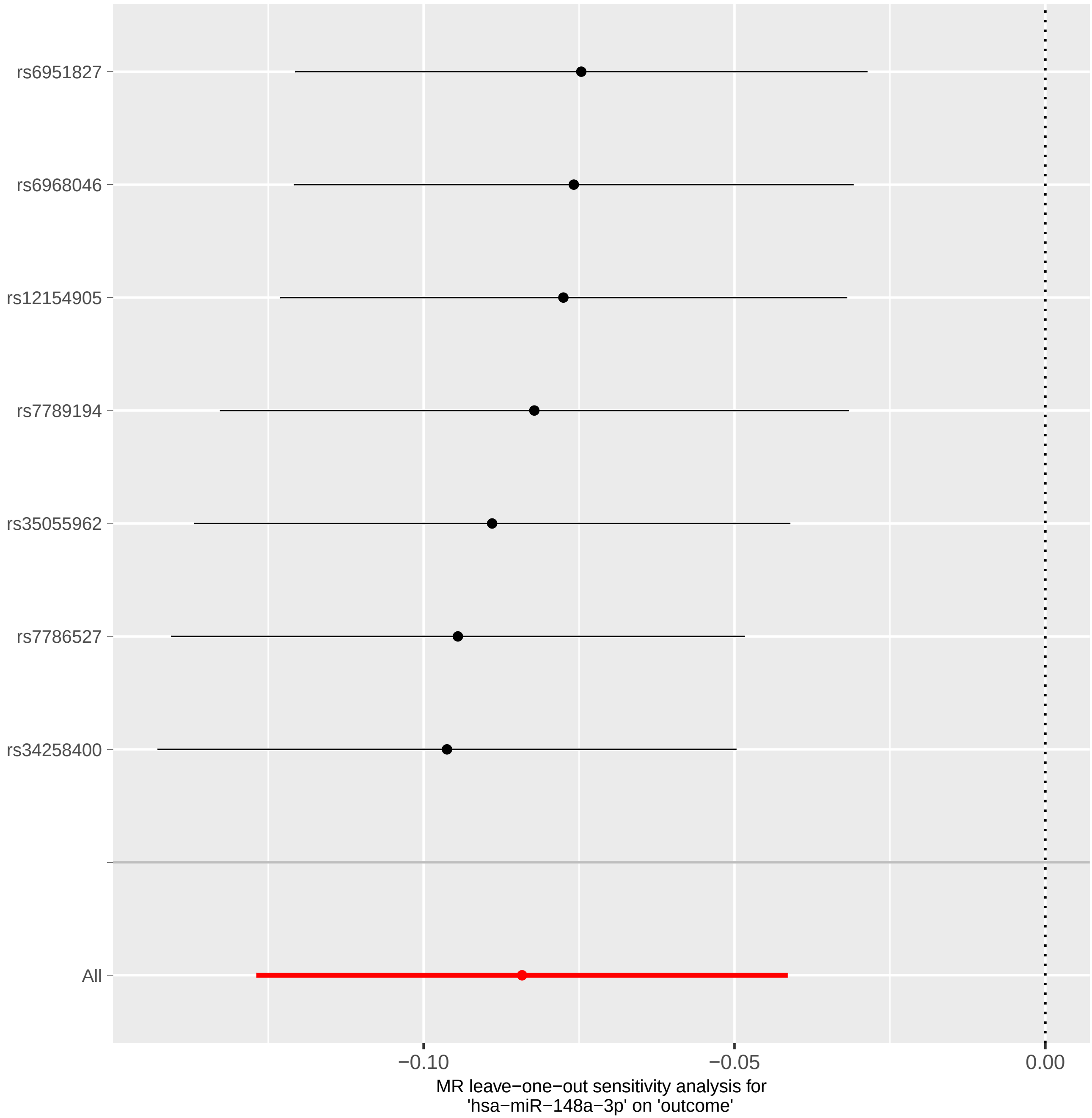
rs6951827
rs6968046
rs12154905
rs7789194
rs35055962
rs7786527
rs34258400
All
−0.10
−0.05
0.00
MR leave−one−out sensitivity analysis for
'hsa−miR−148a−3p' on 'outcome'
