# Supplementary material for: Identifying Putative Causal Links between MicroRNAs and Severe COVID-19 Using Mendelian Randomization
Source: Cells. 2021 Dec 11;10(12):3504. doi: 10.3390/cells10123504 (PMC8700362; doi:10.3390/cells10123504)
Supplement: Supplementary file 1 [file cells-10-03504-s001.zip › Supplementary Figures.zip!/A2_LOO/hsa-miR-152A2.pdf]

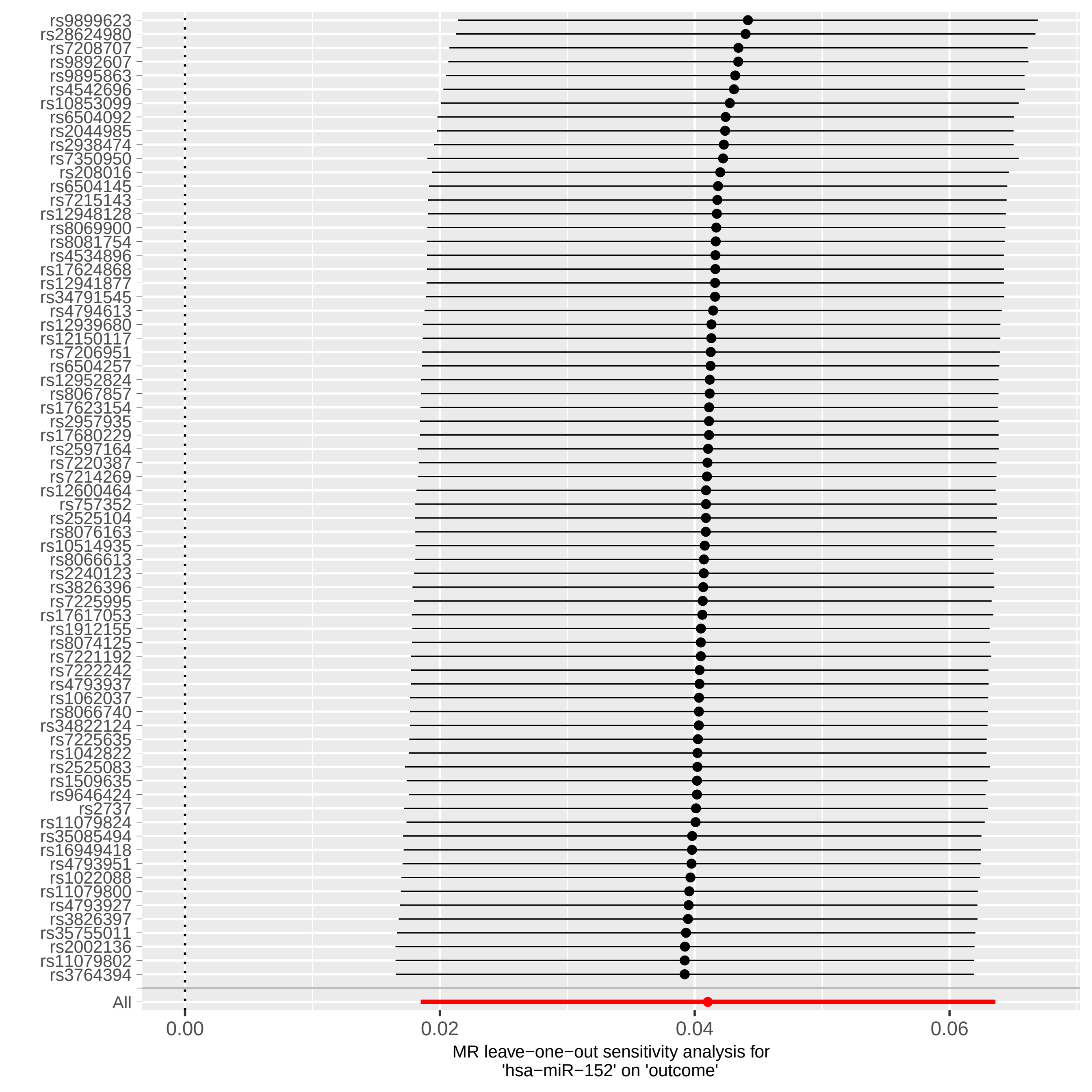

rs9899623
rs28624980
rs7208707
rs9892607
rs9895863
rs4542696
rs10853099
rs6504092
rs2044985
rs2938474
rs7350950
rs208016
rs6504145
rs7215143
rs12948128
rs8069900
rs8081754
rs4534896
rs17624868
rs12941877
rs34791545
rs4794613
rs12939680
rs12150117
rs7206951
rs6504257
rs12952824
rs8067857
rs17623154
rs2957935
rs17680229
rs2597164
rs7220387
rs7214269
rs12600464
rs757352
rs2525104
rs8076163
rs10514935
rs8066613
rs2240123
rs3826396
rs7225995
rs17617053
rs1912155
rs8074125
rs7221192
rs7222242
rs4793937
rs1062037
rs8066740
rs34822124
rs7225635
rs1042822
rs2525083
rs1509635
rs9646424
rs2737
rs11079824
rs35085494
rs16949418
rs4793951
rs1022088
rs11079800
rs4793927
rs3826397
rs35755011
rs2002136
rs11079802
rs3764394
All
0.00
0.02
0.04
0.06
MR leave−one−out sensitivity analysis for
'hsa−miR−152' on 'outcome'
