# Supplementary material for: Identifying Putative Causal Links between MicroRNAs and Severe COVID-19 Using Mendelian Randomization
Source: Cells. 2021 Dec 11;10(12):3504. doi: 10.3390/cells10123504 (PMC8700362; doi:10.3390/cells10123504)
Supplement: Supplementary file 1 [file cells-10-03504-s001.zip › Supplementary Figures.zip!/A2_LOO/hsa-miR-196b-5pA2.pdf]

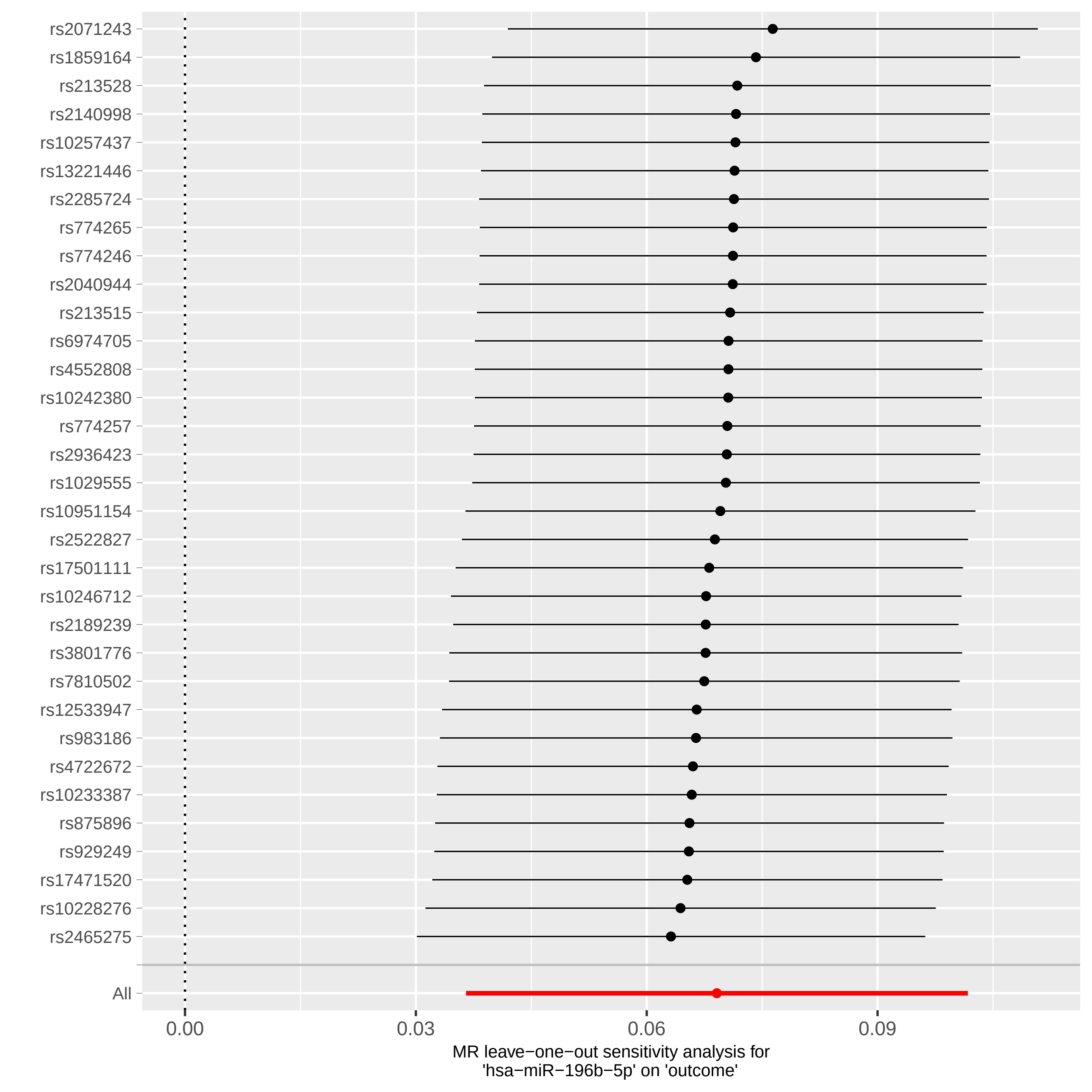

rs2071243
rs1859164
rs213528
rs2140998
rs10257437
rs13221446
rs2285724
rs774265
rs774246
rs2040944
rs213515
rs6974705
rs4552808
rs10242380
rs774257
rs2936423
rs1029555
rs10951154
rs2522827
rs17501111
rs10246712
rs2189239
rs3801776
rs7810502
rs12533947
rs983186
rs4722672
rs10233387
rs875896
rs929249
rs17471520
rs10228276
rs2465275
All
0.00
0.03
0.06
0.09
MR leave−one−out sensitivity analysis for
'hsa−miR−196b−5p' on 'outcome'
