# Supplementary material for: Identifying Putative Causal Links between MicroRNAs and Severe COVID-19 Using Mendelian Randomization
Source: Cells. 2021 Dec 11;10(12):3504. doi: 10.3390/cells10123504 (PMC8700362; doi:10.3390/cells10123504)
Supplement: Supplementary file 1 [file cells-10-03504-s001.zip › Supplementary Figures.zip!/A2_LOO/hsa-miR-204-5pA2.pdf]

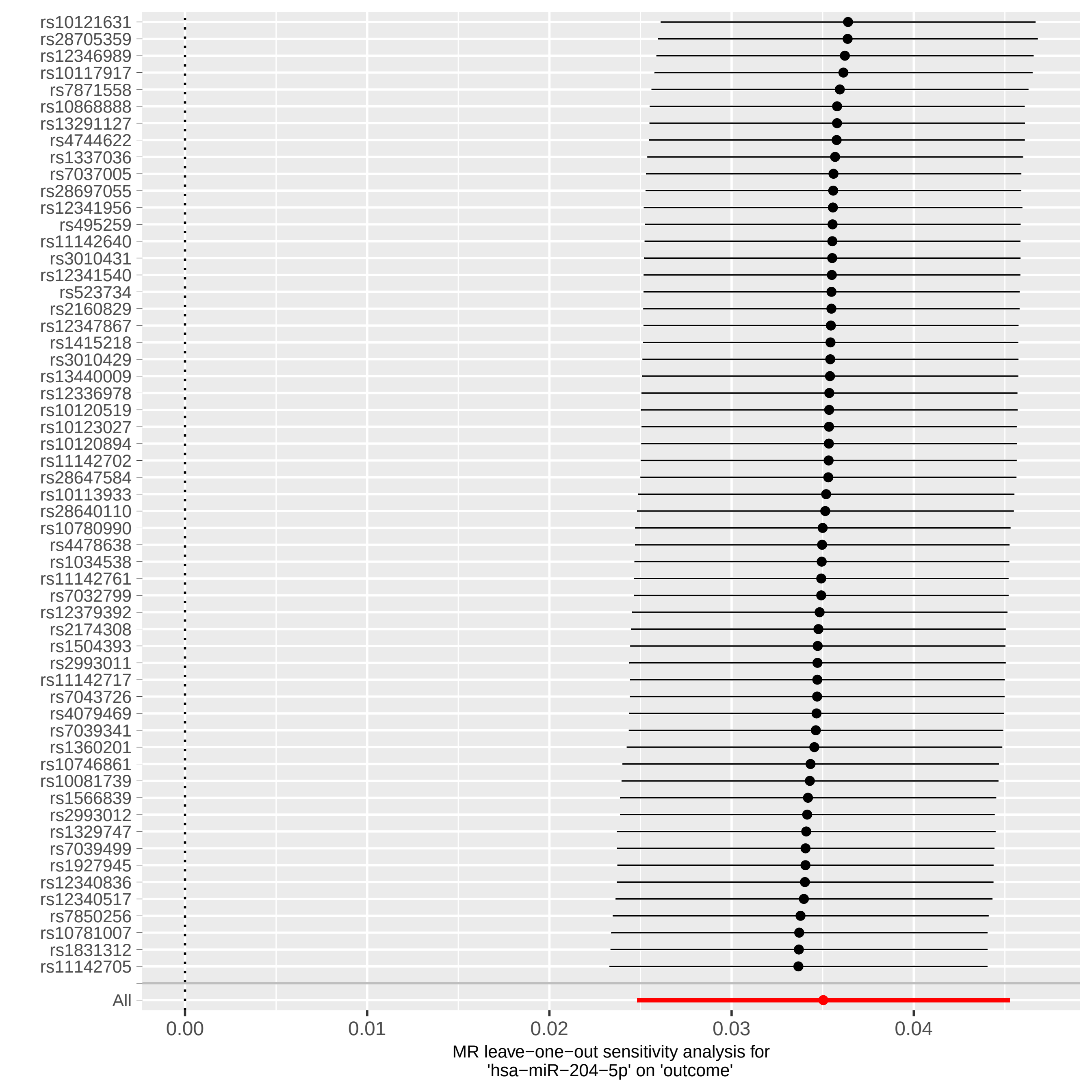
rs10121631
rs28705359
rs12346989
rs10117917
rs7871558
rs10868888
rs13291127
rs4744622
rs1337036
rs7037005
rs28697055
rs12341956
rs495259
rs11142640
rs3010431
rs12341540
rs523734
rs2160829
rs12347867
rs1415218
rs3010429
rs13440009
rs12336978
rs10120519
rs10123027
rs10120894
rs11142702
rs28647584
rs10113933
rs28640110
rs10780990
rs4478638
rs1034538
rs11142761
rs7032799
rs12379392
rs2174308
rs1504393
rs2993011
rs11142717
rs7043726
rs4079469
rs7039341
rs1360201
rs10746861
rs10081739
rs1566839
rs2993012
rs1329747
rs7039499
rs1927945
rs12340836
rs12340517
rs7850256
rs10781007
rs1831312
rs11142705
All
0.00
0.01
0.02
0.03
0.04
MR leave−one−out sensitivity analysis for
'hsa−miR−204−5p' on 'outcome'
