# Supplementary material for: Identifying Putative Causal Links between MicroRNAs and Severe COVID-19 Using Mendelian Randomization
Source: Cells. 2021 Dec 11;10(12):3504. doi: 10.3390/cells10123504 (PMC8700362; doi:10.3390/cells10123504)
Supplement: Supplementary file 1 [file cells-10-03504-s001.zip › Supplementary Figures.zip!/A2_LOO/hsa-miR-30a-3pA2.pdf]

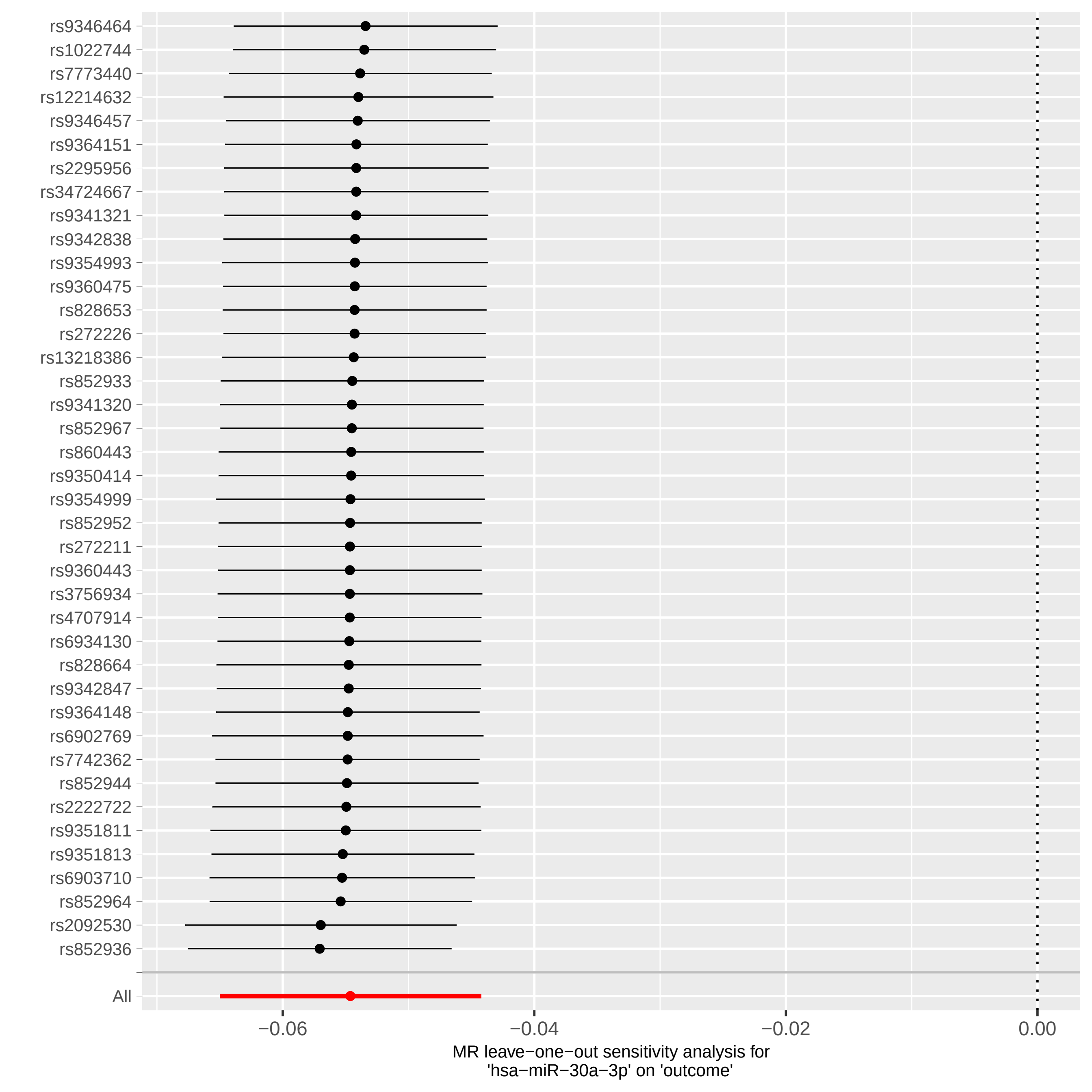

rs9346464
rs1022744
rs7773440
rs12214632
rs9346457
rs9364151
rs2295956
rs34724667
rs9341321
rs9342838
rs9354993
rs9360475
rs828653
rs272226
rs13218386
rs852933
rs9341320
rs852967
rs860443
rs9350414
rs9354999
rs852952
rs272211
rs9360443
rs3756934
rs4707914
rs6934130
rs828664
rs9342847
rs9364148
rs6902769
rs7742362
rs852944
rs2222722
rs9351811
rs9351813
rs6903710
rs852964
rs2092530
rs852936
All
−0.06
−0.04
−0.02
0.00
MR leave−one−out sensitivity analysis for
'hsa−miR−30a−3p' on 'outcome'
