# Supplementary material for: Identifying Putative Causal Links between MicroRNAs and Severe COVID-19 Using Mendelian Randomization
Source: Cells. 2021 Dec 11;10(12):3504. doi: 10.3390/cells10123504 (PMC8700362; doi:10.3390/cells10123504)
Supplement: Supplementary file 1 [file cells-10-03504-s001.zip › Supplementary Figures.zip!/A2_LOO/hsa-miR-744-5pA2.pdf]

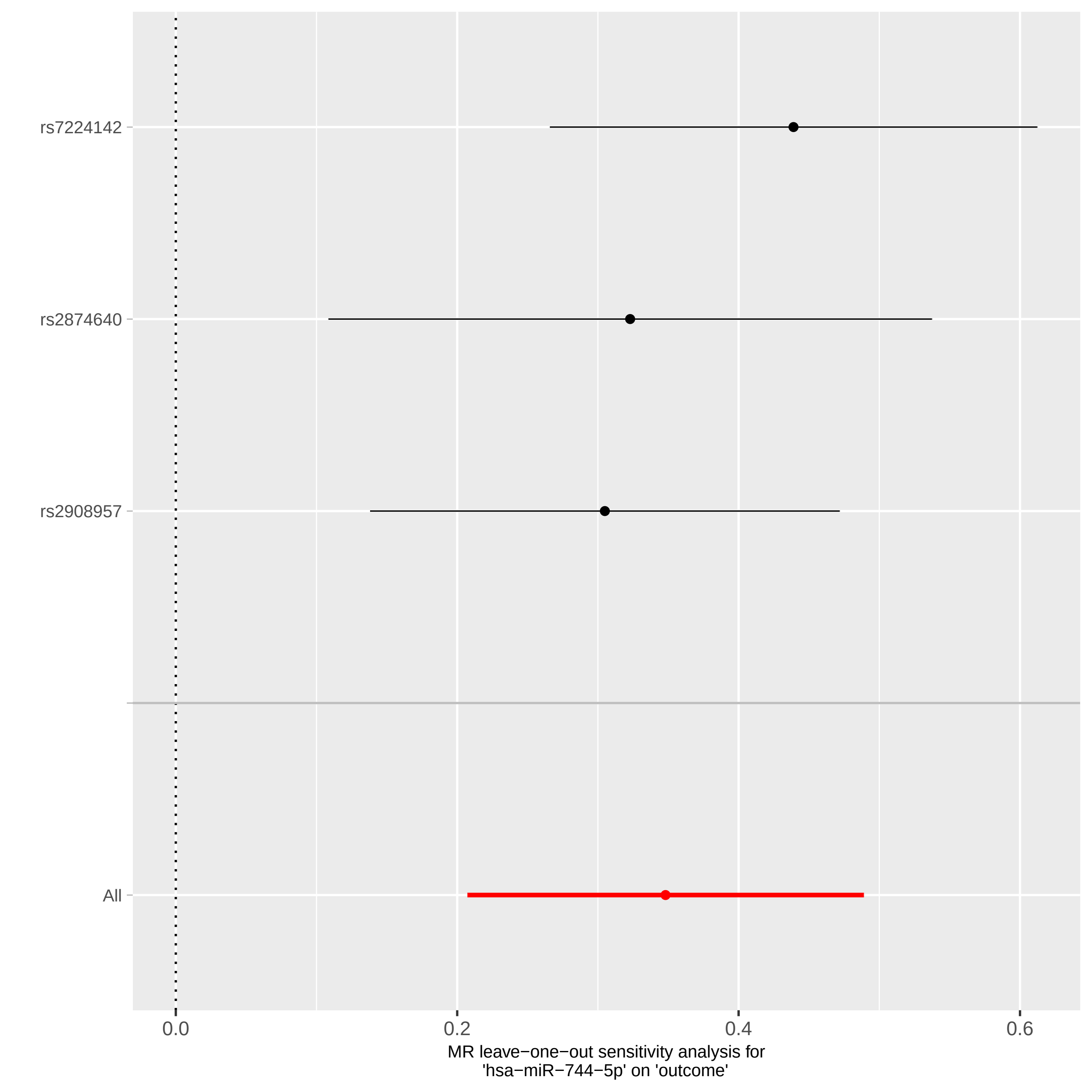

rs7224142
rs2874640
rs2908957
All
0.0
0.2
0.4
0.6
MR leave−one−out sensitivity analysis for
'hsa−miR−744−5p' on 'outcome'
