# Supplementary material for: Identifying Putative Causal Links between MicroRNAs and Severe COVID-19 Using Mendelian Randomization
Source: Cells. 2021 Dec 11;10(12):3504. doi: 10.3390/cells10123504 (PMC8700362; doi:10.3390/cells10123504)
Supplement: Supplementary file 1 [file cells-10-03504-s001.zip › Supplementary Figures.zip!/A2_LOO/hsa-miR-885-5pA2.pdf]

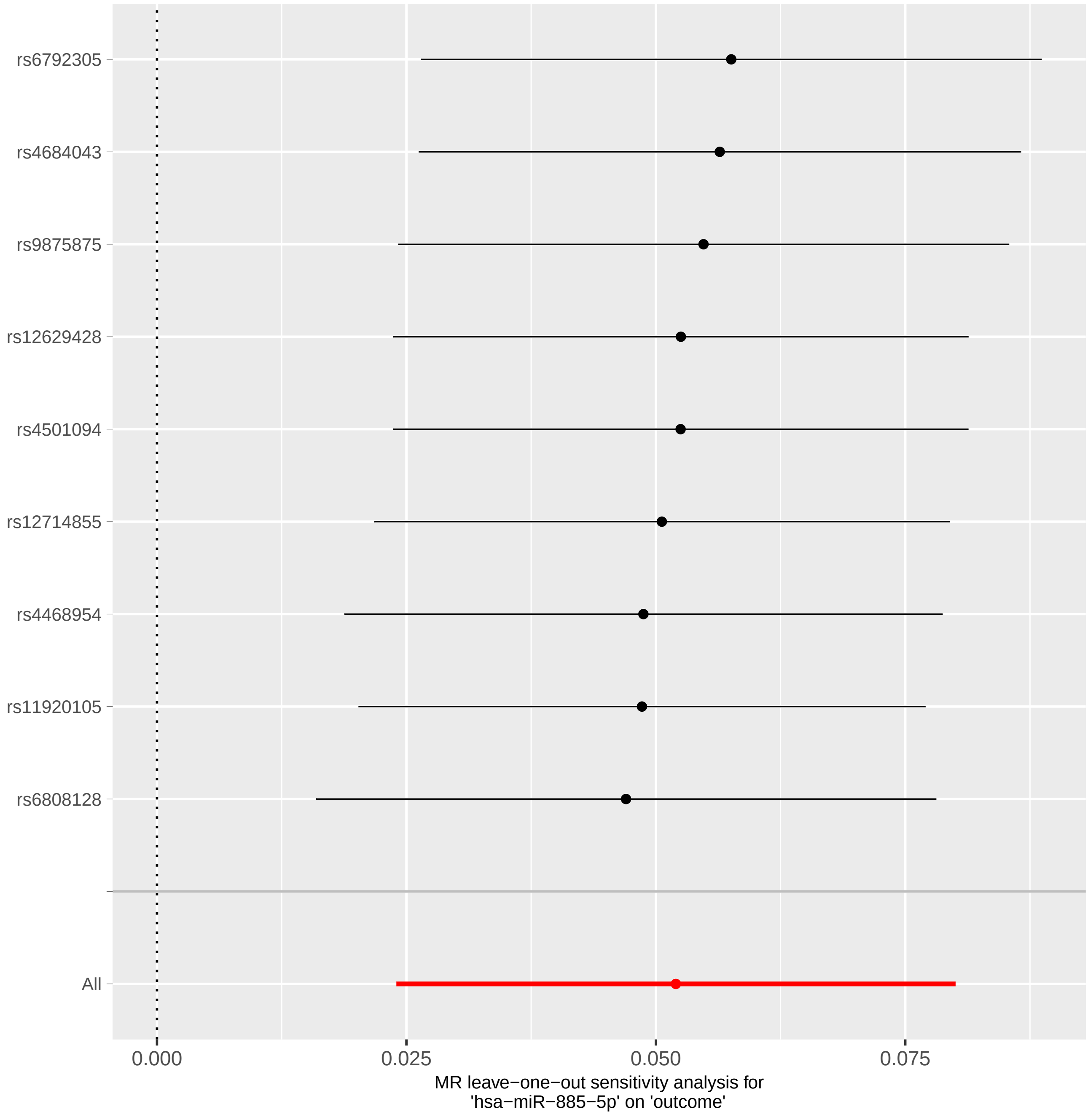
rs6792305
rs4684043
rs9875875
rs12629428
rs4501094
rs12714855
rs4468954
rs11920105
rs6808128
All
0.000
0.025
0.050
0.075
MR leave−one−out sensitivity analysis for
'hsa−miR−885−5p' on 'outcome'
