# Supplementary material for: Identifying Putative Causal Links between MicroRNAs and Severe COVID-19 Using Mendelian Randomization
Source: Cells. 2021 Dec 11;10(12):3504. doi: 10.3390/cells10123504 (PMC8700362; doi:10.3390/cells10123504)
Supplement: Supplementary file 1 [file cells-10-03504-s001.zip › Supplementary Figures.zip!/B1_LOO/hsa-miR-1303B1.pdf]

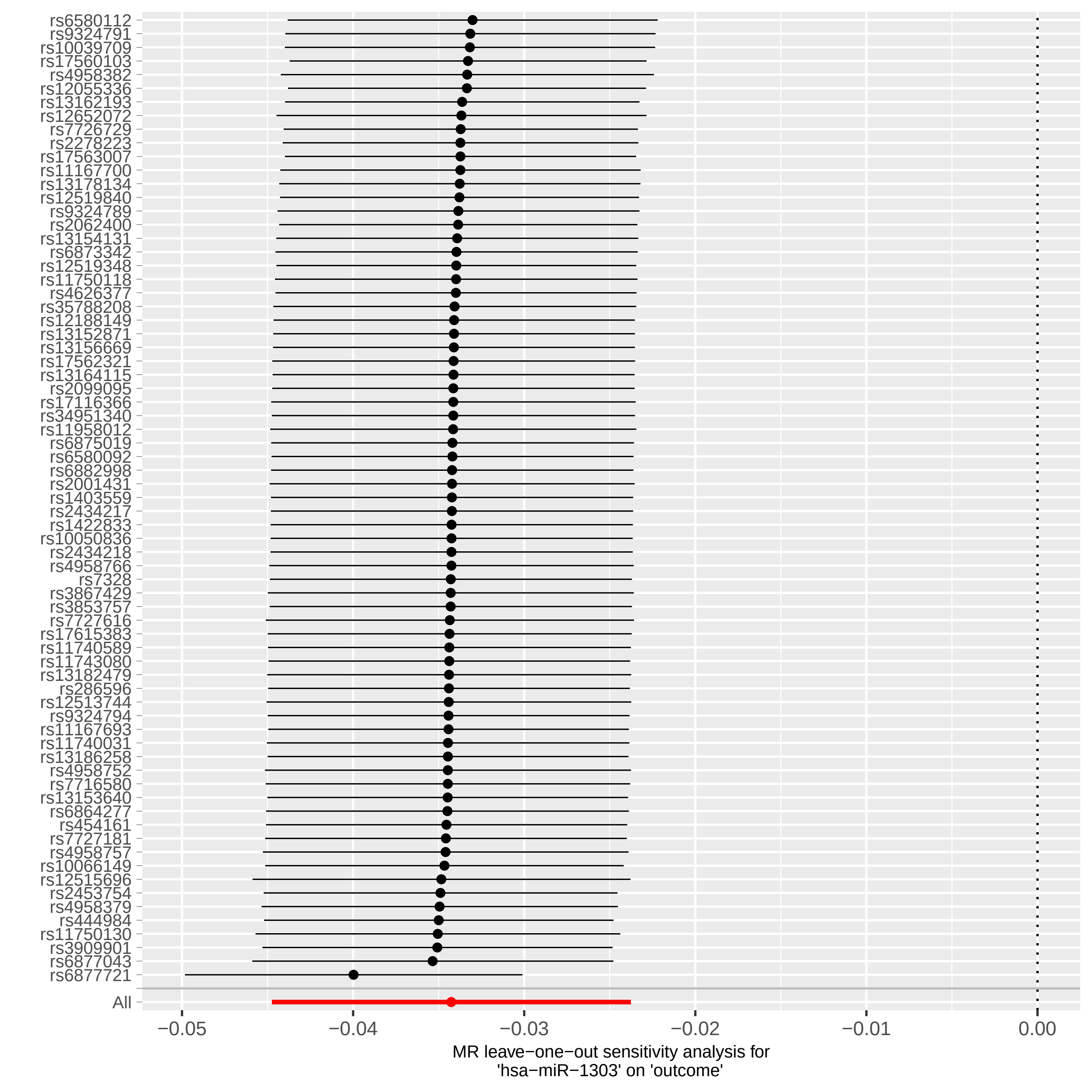
rs6580112
rs9324791
rs10039709
rs17560103
rs4958382
rs12055336
rs13162193
rs12652072
rs7726729
rs2278223
rs17563007
rs11167700
rs13178134
rs12519840
rs9324789
rs2062400
rs13154131
rs6873342
rs12519348
rs11750118
rs4626377
rs35788208
rs12188149
rs13152871
rs13156669
rs17562321
rs13164115
rs2099095
rs17116366
rs34951340
rs11958012
rs6875019
rs6580092
rs6882998
rs2001431
rs1403559
rs2434217
rs1422833
rs10050836
rs2434218
rs4958766
rs7328
rs3867429
rs3853757
rs7727616
rs17615383
rs11740589
rs11743080
rs13182479
rs286596
rs12513744
rs9324794
rs11167693
rs11740031
rs13186258
rs4958752
rs7716580
rs13153640
rs6864277
rs454161
rs7727181
rs4958757
rs10066149
rs12515696
rs2453754
rs4958379
rs444984
rs11750130
rs3909901
rs6877043
rs6877721
All
−0.05
−0.04
−0.03
−0.02
−0.01
0.00
MR leave−one−out sensitivity analysis for
'hsa−miR−1303' on 'outcome'
