# Supplementary material for: Identifying Putative Causal Links between MicroRNAs and Severe COVID-19 Using Mendelian Randomization
Source: Cells. 2021 Dec 11;10(12):3504. doi: 10.3390/cells10123504 (PMC8700362; doi:10.3390/cells10123504)
Supplement: Supplementary file 1 [file cells-10-03504-s001.zip › Supplementary Figures.zip!/B1_LOO/hsa-miR-30a-3pB1.pdf]

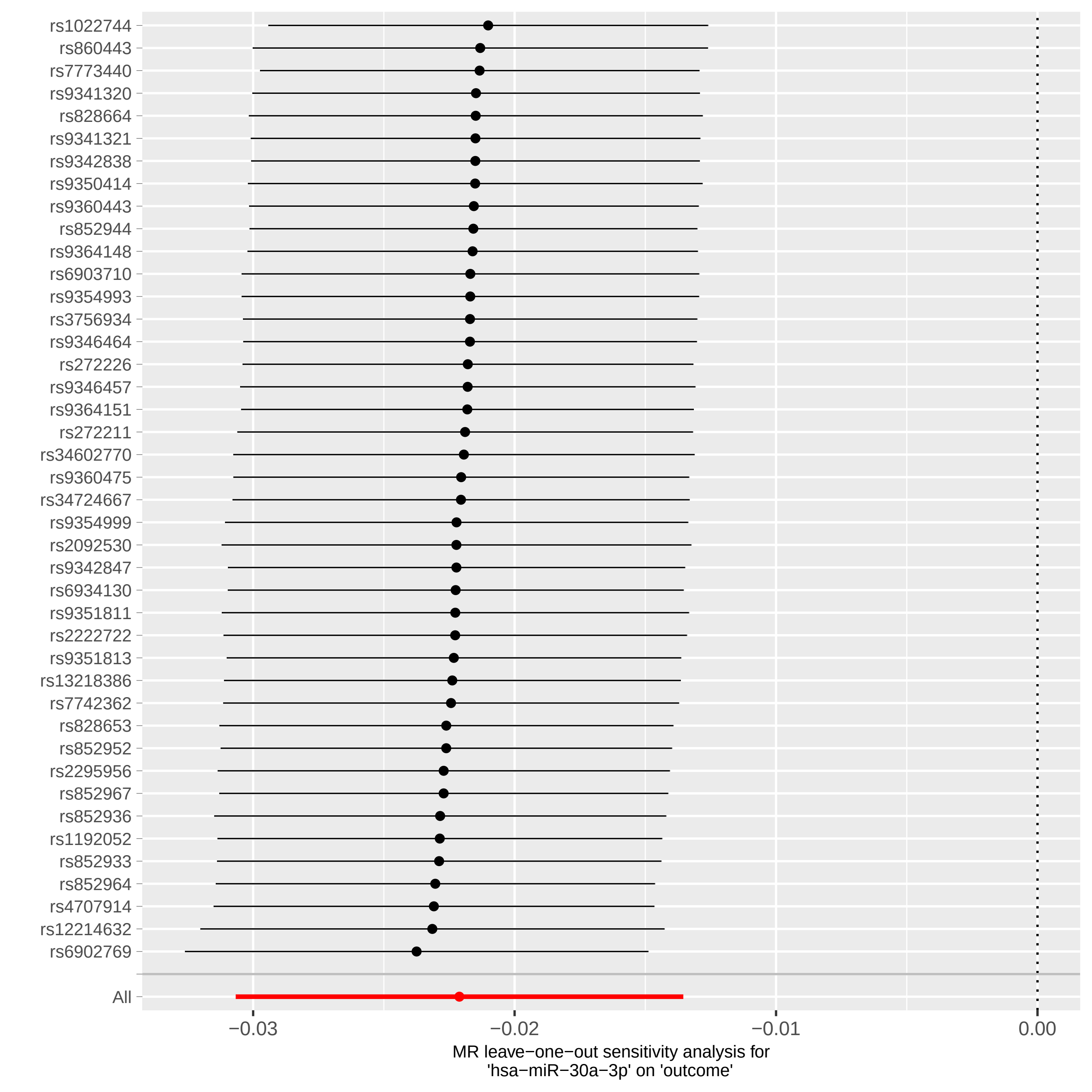
rs1022744
rs860443
rs7773440
rs9341320
rs828664
rs9341321
rs9342838
rs9350414
rs9360443
rs852944
rs9364148
rs6903710
rs9354993
rs3756934
rs9346464
rs272226
rs9346457
rs9364151
rs272211
rs34602770
rs9360475
rs34724667
rs9354999
rs2092530
rs9342847
rs6934130
rs9351811
rs2222722
rs9351813
rs13218386
rs7742362
rs828653
rs852952
rs2295956
rs852967
rs852936
rs1192052
rs852933
rs852964
rs4707914
rs12214632
rs6902769
All
−0.03
−0.02
−0.01
0.00
MR leave−one−out sensitivity analysis for
'hsa−miR−30a−3p' on 'outcome'
