# Supplementary material for: Identifying Putative Causal Links between MicroRNAs and Severe COVID-19 Using Mendelian Randomization
Source: Cells. 2021 Dec 11;10(12):3504. doi: 10.3390/cells10123504 (PMC8700362; doi:10.3390/cells10123504)
Supplement: Supplementary file 1 [file cells-10-03504-s001.zip › Supplementary Figures.zip!/B1_LOO/hsa-miR-339-3pB1.pdf]

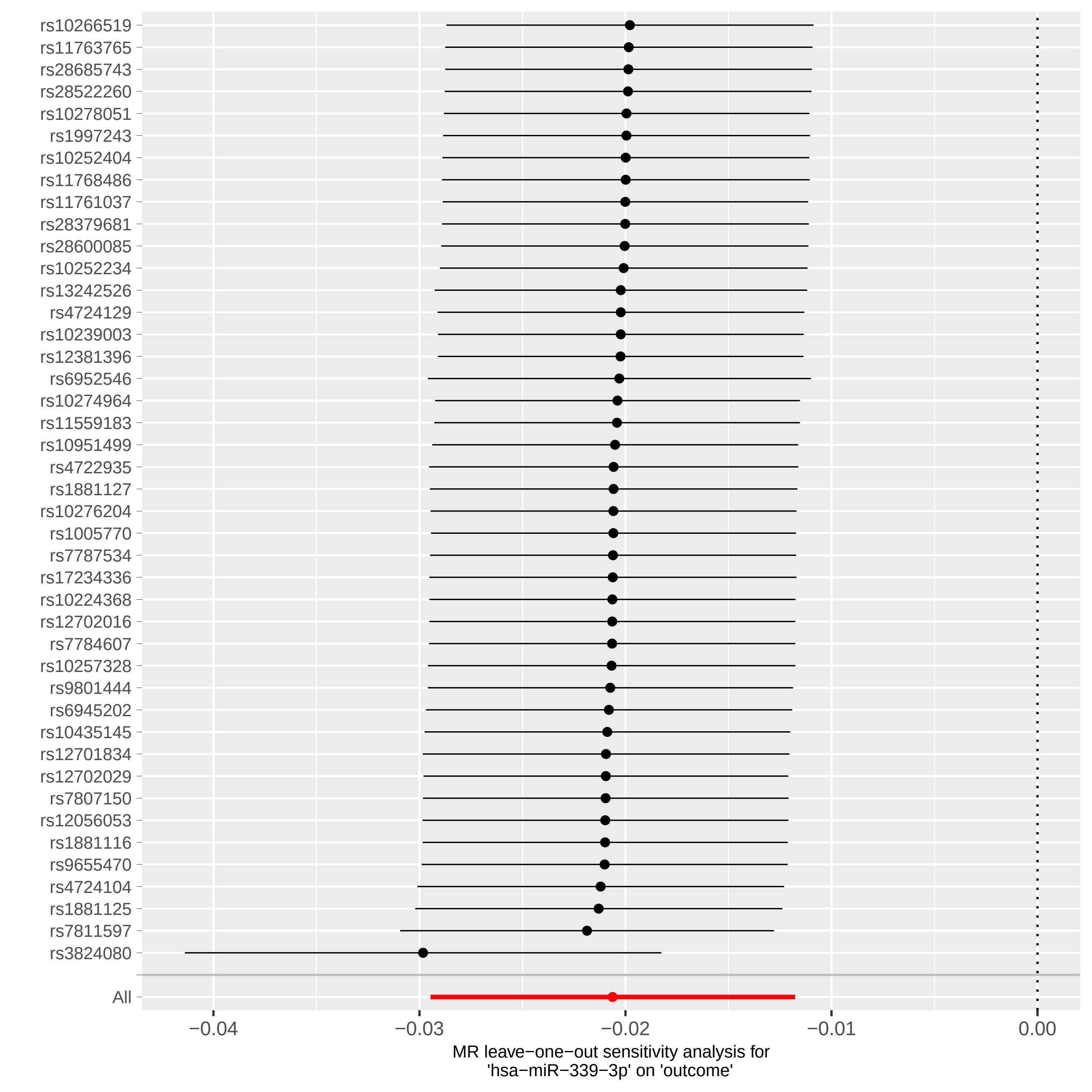
rs10266519
rs11763765
rs28685743
rs28522260
rs10278051
rs1997243
rs10252404
rs11768486
rs11761037
rs28379681
rs28600085
rs10252234
rs13242526
rs4724129
rs10239003
rs12381396
rs6952546
rs10274964
rs11559183
rs10951499
rs4722935
rs1881127
rs10276204
rs1005770
rs7787534
rs17234336
rs10224368
rs12702016
rs7784607
rs10257328
rs9801444
rs6945202
rs10435145
rs12701834
rs12702029
rs7807150
rs12056053
rs1881116
rs9655470
rs4724104
rs1881125
rs7811597
rs3824080
All
−0.04
−0.03
−0.02
−0.01
0.00
MR leave−one−out sensitivity analysis for
'hsa−miR−339−3p' on 'outcome'
