# Supplementary material for: Identifying Putative Causal Links between MicroRNAs and Severe COVID-19 Using Mendelian Randomization
Source: Cells. 2021 Dec 11;10(12):3504. doi: 10.3390/cells10123504 (PMC8700362; doi:10.3390/cells10123504)
Supplement: Supplementary file 1 [file cells-10-03504-s001.zip › Supplementary Figures.zip!/B1_LOO/hsa-miR-339-5pB1.pdf]

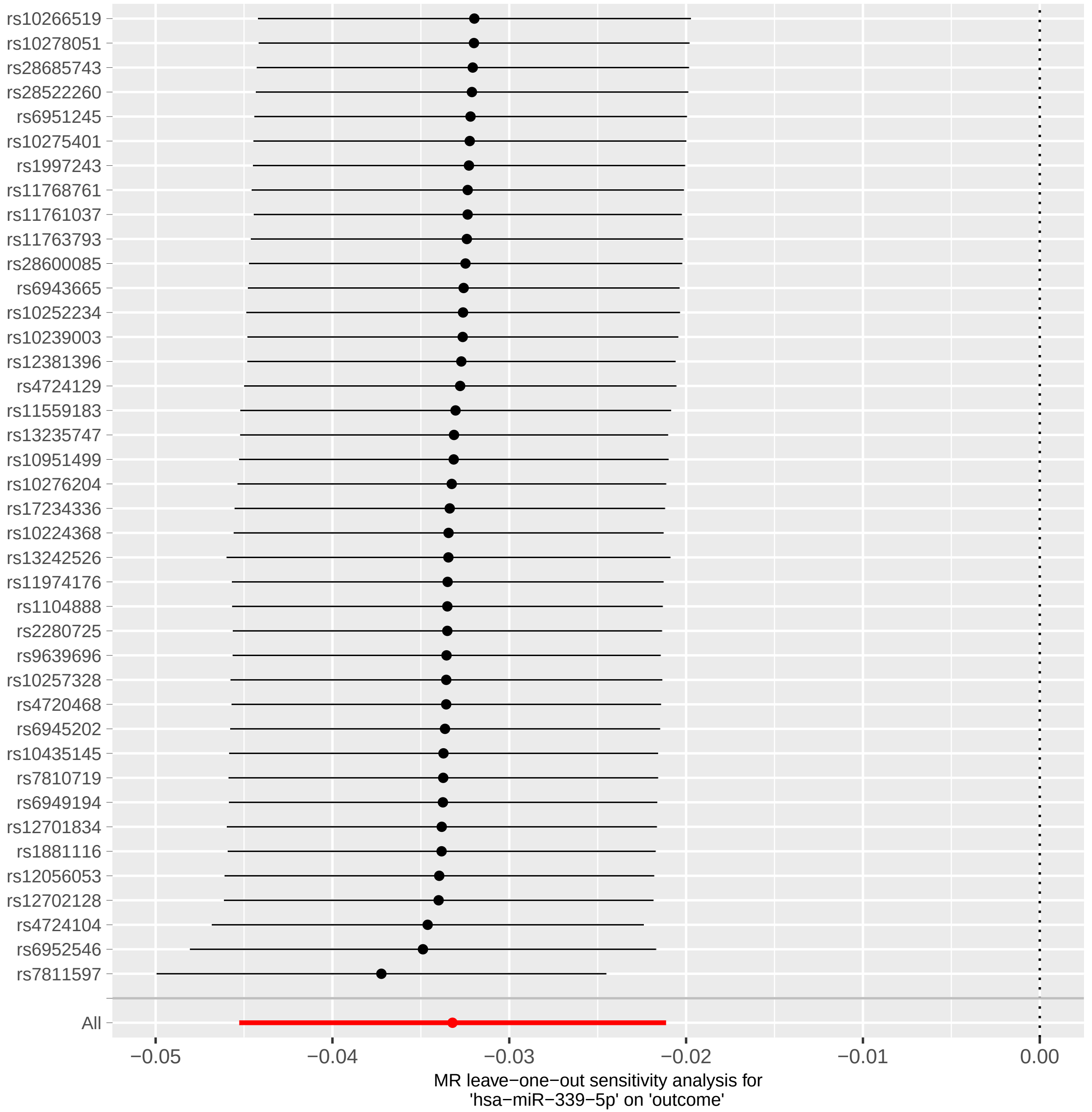
rs10266519
rs10278051
rs28685743
rs28522260
rs6951245
rs10275401
rs1997243
rs11768761
rs11761037
rs11763793
rs28600085
rs6943665
rs10252234
rs10239003
rs12381396
rs4724129
rs11559183
rs13235747
rs10951499
rs10276204
rs17234336
rs10224368
rs13242526
rs11974176
rs1104888
rs2280725
rs9639696
rs10257328
rs4720468
rs6945202
rs10435145
rs7810719
rs6949194
rs12701834
rs1881116
rs12056053
rs12702128
rs4724104
rs6952546
rs7811597
All
−0.05
−0.04
−0.03
−0.02
−0.01
0.00
MR leave−one−out sensitivity analysis for
'hsa−miR−339−5p' on 'outcome'
