# Supplementary material for: Identifying Putative Causal Links between MicroRNAs and Severe COVID-19 Using Mendelian Randomization
Source: Cells. 2021 Dec 11;10(12):3504. doi: 10.3390/cells10123504 (PMC8700362; doi:10.3390/cells10123504)
Supplement: Supplementary file 1 [file cells-10-03504-s001.zip › Supplementary Figures.zip!/B2_LOO/hsa-miR-130a-3pB2.pdf]

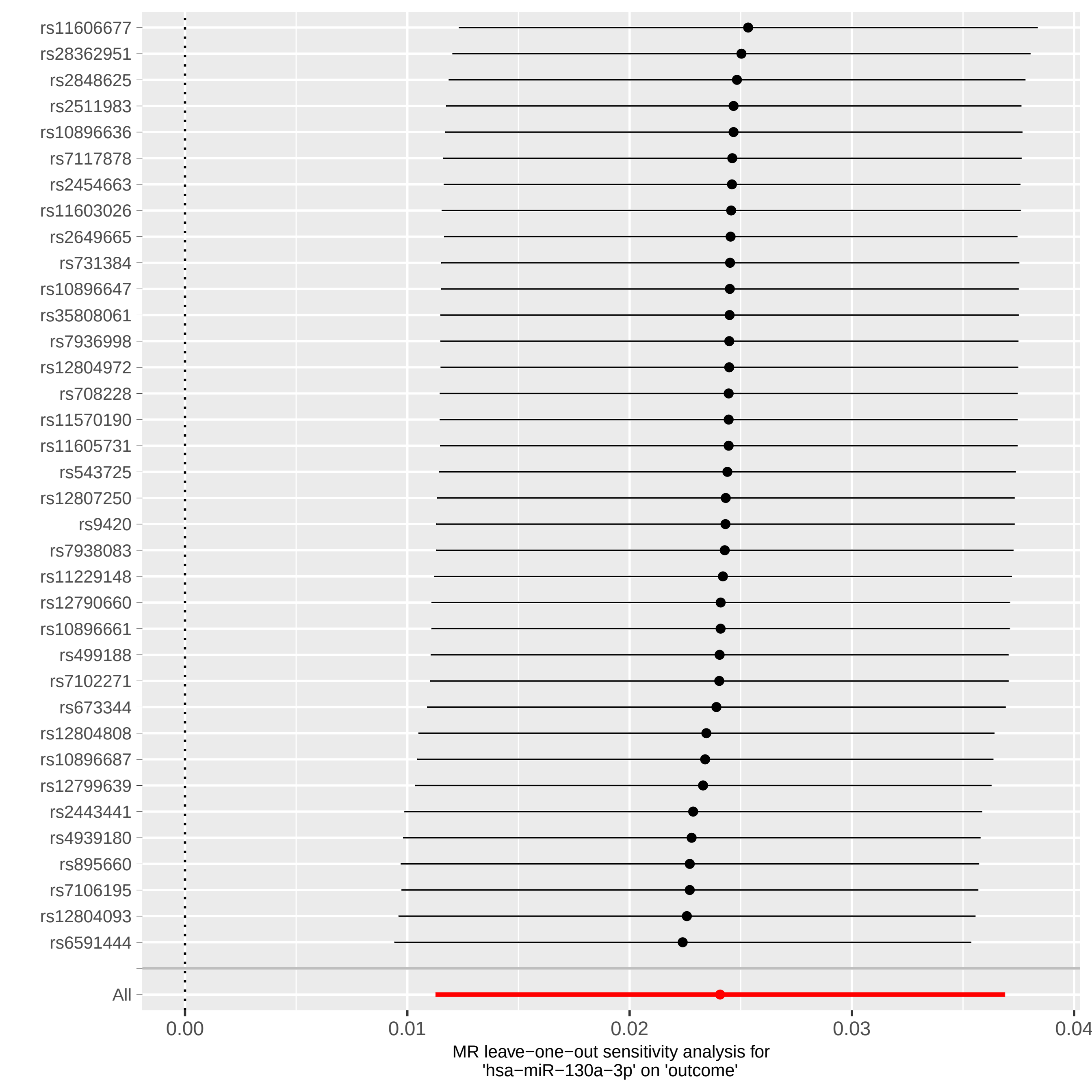
rs11606677
rs28362951
rs2848625
rs2511983
rs10896636
rs7117878
rs2454663
rs11603026
rs2649665
rs731384
rs10896647
rs35808061
rs7936998
rs12804972
rs708228
rs11570190
rs11605731
rs543725
rs12807250
rs9420
rs7938083
rs11229148
rs12790660
rs10896661
rs499188
rs7102271
rs673344
rs12804808
rs10896687
rs12799639
rs2443441
rs4939180
rs895660
rs7106195
rs12804093
rs6591444
All
0.00
0.01
0.02
0.03
0.04
MR leave−one−out sensitivity analysis for
'hsa−miR−130a−3p' on 'outcome'
