# Supplementary material for: Identifying Putative Causal Links between MicroRNAs and Severe COVID-19 Using Mendelian Randomization
Source: Cells. 2021 Dec 11;10(12):3504. doi: 10.3390/cells10123504 (PMC8700362; doi:10.3390/cells10123504)
Supplement: Supplementary file 1 [file cells-10-03504-s001.zip › Supplementary Figures.zip!/B2_LOO/hsa-miR-135a-5pB2.pdf]

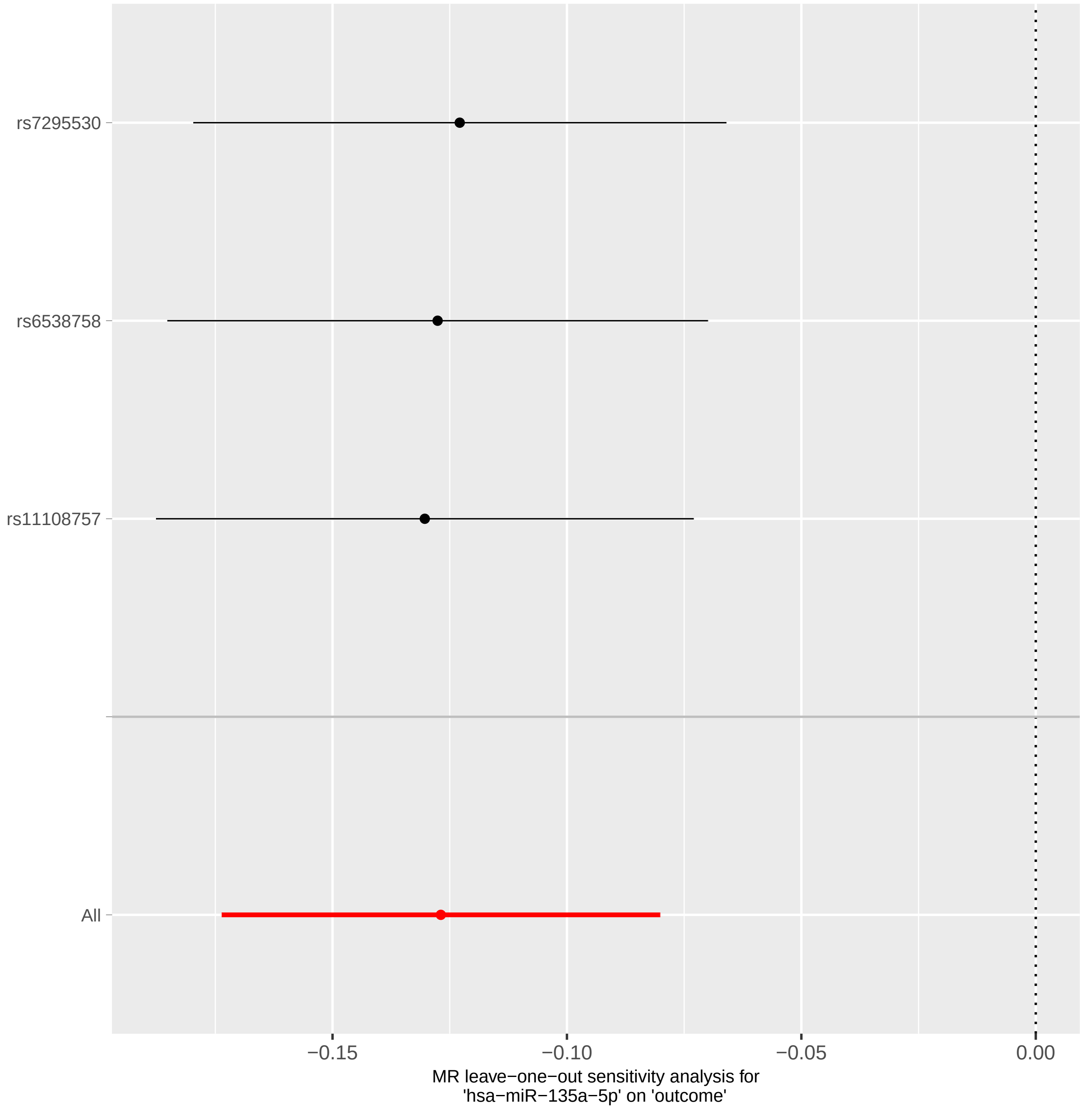
rs7295530
rs6538758
rs11108757
All
−0.15
−0.10
−0.05
0.00
MR leave−one−out sensitivity analysis for
'hsa−miR−135a−5p' on 'outcome'
