# Supplementary material for: Identifying Putative Causal Links between MicroRNAs and Severe COVID-19 Using Mendelian Randomization
Source: Cells. 2021 Dec 11;10(12):3504. doi: 10.3390/cells10123504 (PMC8700362; doi:10.3390/cells10123504)
Supplement: Supplementary file 1 [file cells-10-03504-s001.zip › Supplementary Figures.zip!/B2_LOO/hsa-miR-139-5pB2.pdf]

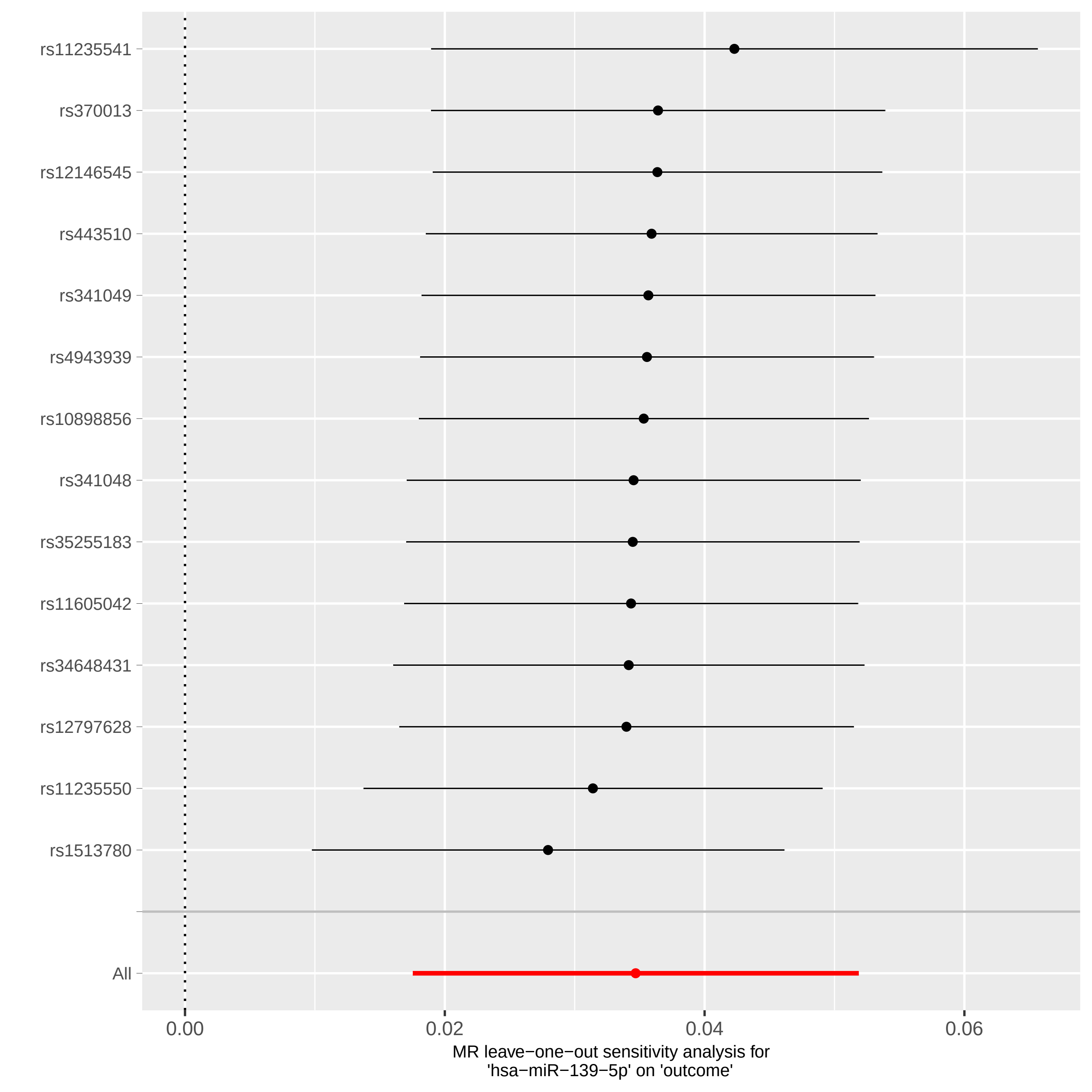

rs11235541
rs370013
rs12146545
rs443510
rs341049
rs4943939
rs10898856
rs341048
rs35255183
rs11605042
rs34648431
rs12797628
rs11235550
rs1513780
All
0.00
0.02
0.04
0.06
MR leave−one−out sensitivity analysis for
'hsa−miR−139−5p' on 'outcome'
