# Supplementary material for: Identifying Putative Causal Links between MicroRNAs and Severe COVID-19 Using Mendelian Randomization
Source: Cells. 2021 Dec 11;10(12):3504. doi: 10.3390/cells10123504 (PMC8700362; doi:10.3390/cells10123504)
Supplement: Supplementary file 1 [file cells-10-03504-s001.zip › Supplementary Figures.zip!/B2_LOO/hsa-miR-183-3pB2.pdf]

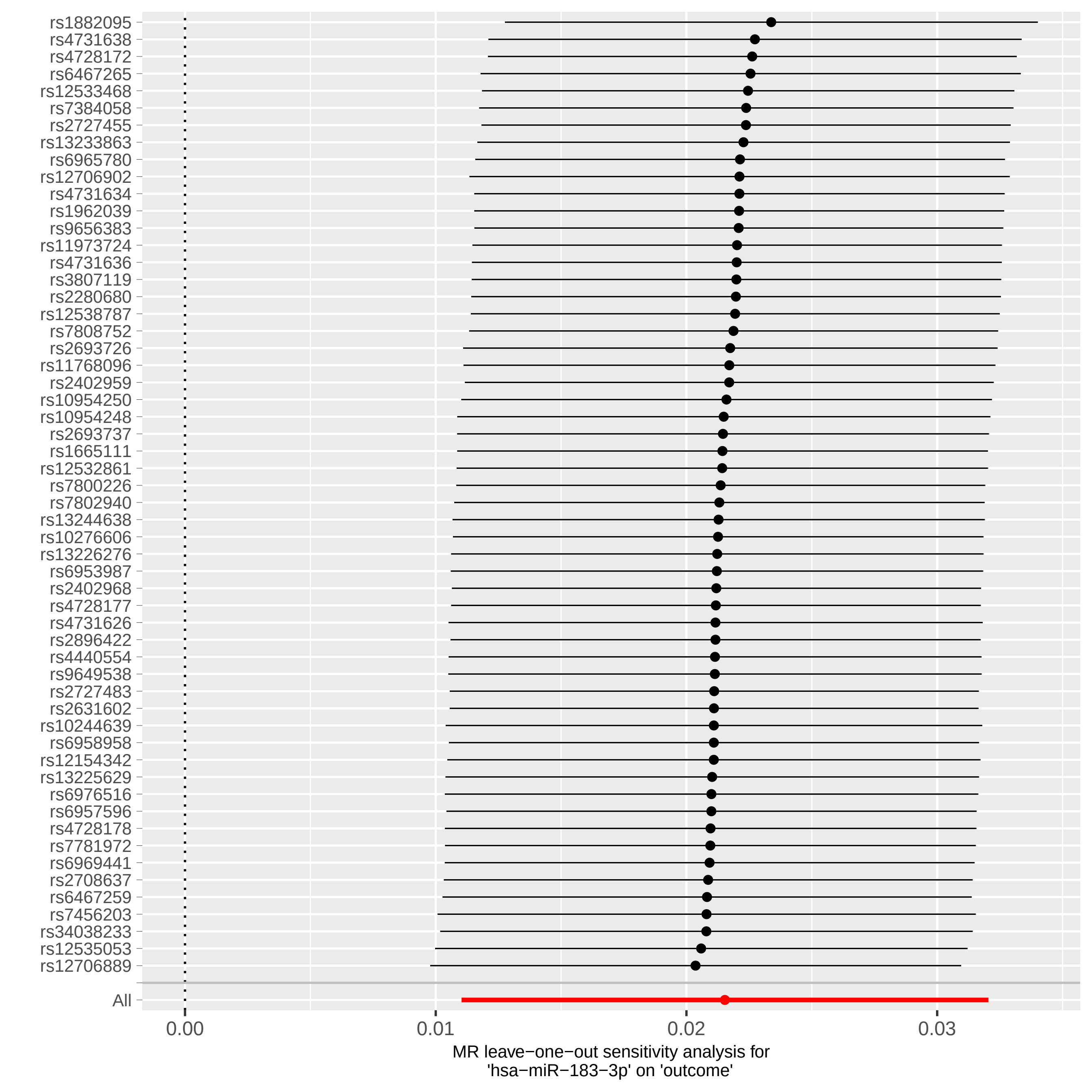
rs1882095
rs4731638
rs4728172
rs6467265
rs12533468
rs7384058
rs2727455
rs13233863
rs6965780
rs12706902
rs4731634
rs1962039
rs9656383
rs11973724
rs4731636
rs3807119
rs2280680
rs12538787
rs7808752
rs2693726
rs11768096
rs2402959
rs10954250
rs10954248
rs2693737
rs1665111
rs12532861
rs7800226
rs7802940
rs13244638
rs10276606
rs13226276
rs6953987
rs2402968
rs4728177
rs4731626
rs2896422
rs4440554
rs9649538
rs2727483
rs2631602
rs10244639
rs6958958
rs12154342
rs13225629
rs6976516
rs6957596
rs4728178
rs7781972
rs6969441
rs2708637
rs6467259
rs7456203
rs34038233
rs12535053
rs12706889
All
0.00
0.01
0.02
0.03
MR leave−one−out sensitivity analysis for
'hsa−miR−183−3p' on 'outcome'
