# Supplementary material for: Identifying Putative Causal Links between MicroRNAs and Severe COVID-19 Using Mendelian Randomization
Source: Cells. 2021 Dec 11;10(12):3504. doi: 10.3390/cells10123504 (PMC8700362; doi:10.3390/cells10123504)
Supplement: Supplementary file 1 [file cells-10-03504-s001.zip › Supplementary Figures.zip!/B2_LOO/hsa-miR-26b-5pB2.pdf]

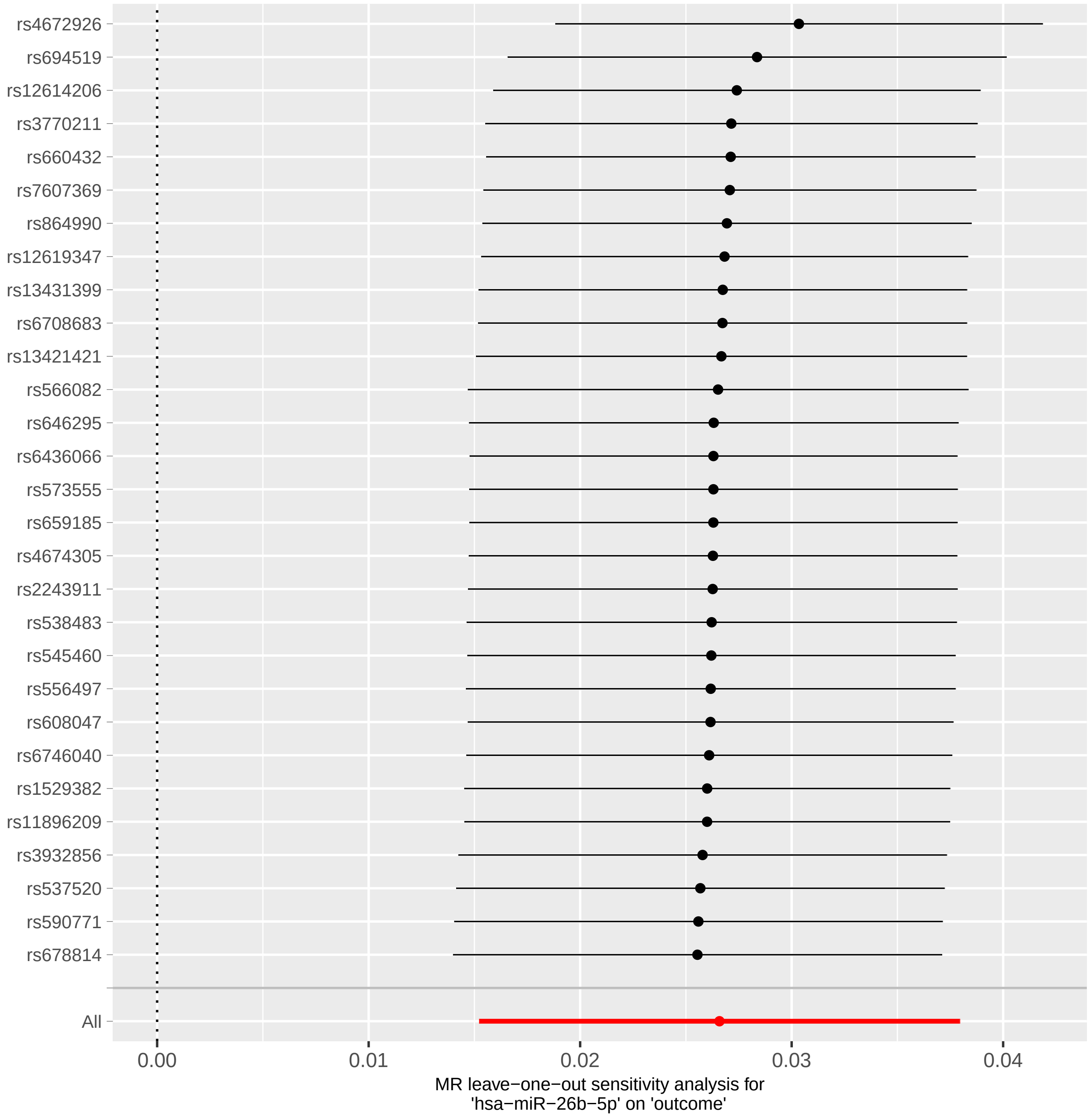
rs4672926
rs694519
rs12614206
rs3770211
rs660432
rs7607369
rs864990
rs12619347
rs13431399
rs6708683
rs13421421
rs566082
rs646295
rs6436066
rs573555
rs659185
rs4674305
rs2243911
rs538483
rs545460
rs556497
rs608047
rs6746040
rs1529382
rs11896209
rs3932856
rs537520
rs590771
rs678814
All
0.00
0.01
0.02
0.03
0.04
MR leave−one−out sensitivity analysis for
'hsa−miR−26b−5p' on 'outcome'
