# Supplementary material for: Identifying Putative Causal Links between MicroRNAs and Severe COVID-19 Using Mendelian Randomization
Source: Cells. 2021 Dec 11;10(12):3504. doi: 10.3390/cells10123504 (PMC8700362; doi:10.3390/cells10123504)
Supplement: Supplementary file 1 [file cells-10-03504-s001.zip › Supplementary Figures.zip!/B2_LOO/hsa-miR-30a-3pB2.pdf]

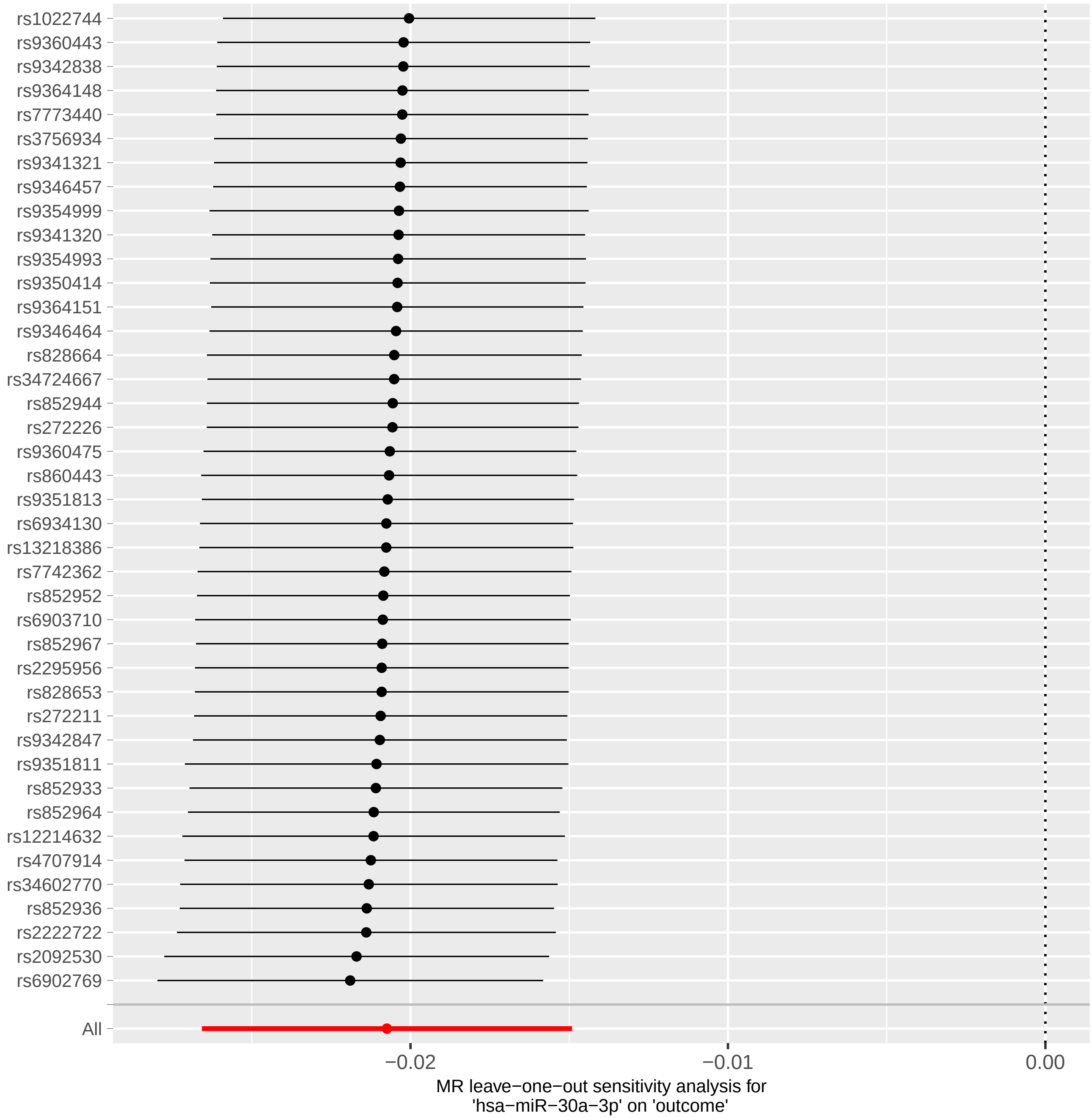
rs1022744
rs9360443
rs9342838
rs9364148
rs7773440
rs3756934
rs9341321
rs9346457
rs9354999
rs9341320
rs9354993
rs9350414
rs9364151
rs9346464
rs828664
rs34724667
rs852944
rs272226
rs9360475
rs860443
rs9351813
rs6934130
rs13218386
rs7742362
rs852952
rs6903710
rs852967
rs2295956
rs828653
rs272211
rs9342847
rs9351811
rs852933
rs852964
rs12214632
rs4707914
rs34602770
rs852936
rs2222722
rs2092530
rs6902769
All
−0.02
−0.01
0.00
MR leave−one−out sensitivity analysis for
'hsa−miR−30a−3p' on 'outcome'
