# Supplementary material for: Identifying Putative Causal Links between MicroRNAs and Severe COVID-19 Using Mendelian Randomization
Source: Cells. 2021 Dec 11;10(12):3504. doi: 10.3390/cells10123504 (PMC8700362; doi:10.3390/cells10123504)
Supplement: Supplementary file 1 [file cells-10-03504-s001.zip › Supplementary Figures.zip!/B2_LOO/hsa-miR-339-5pB2.pdf]

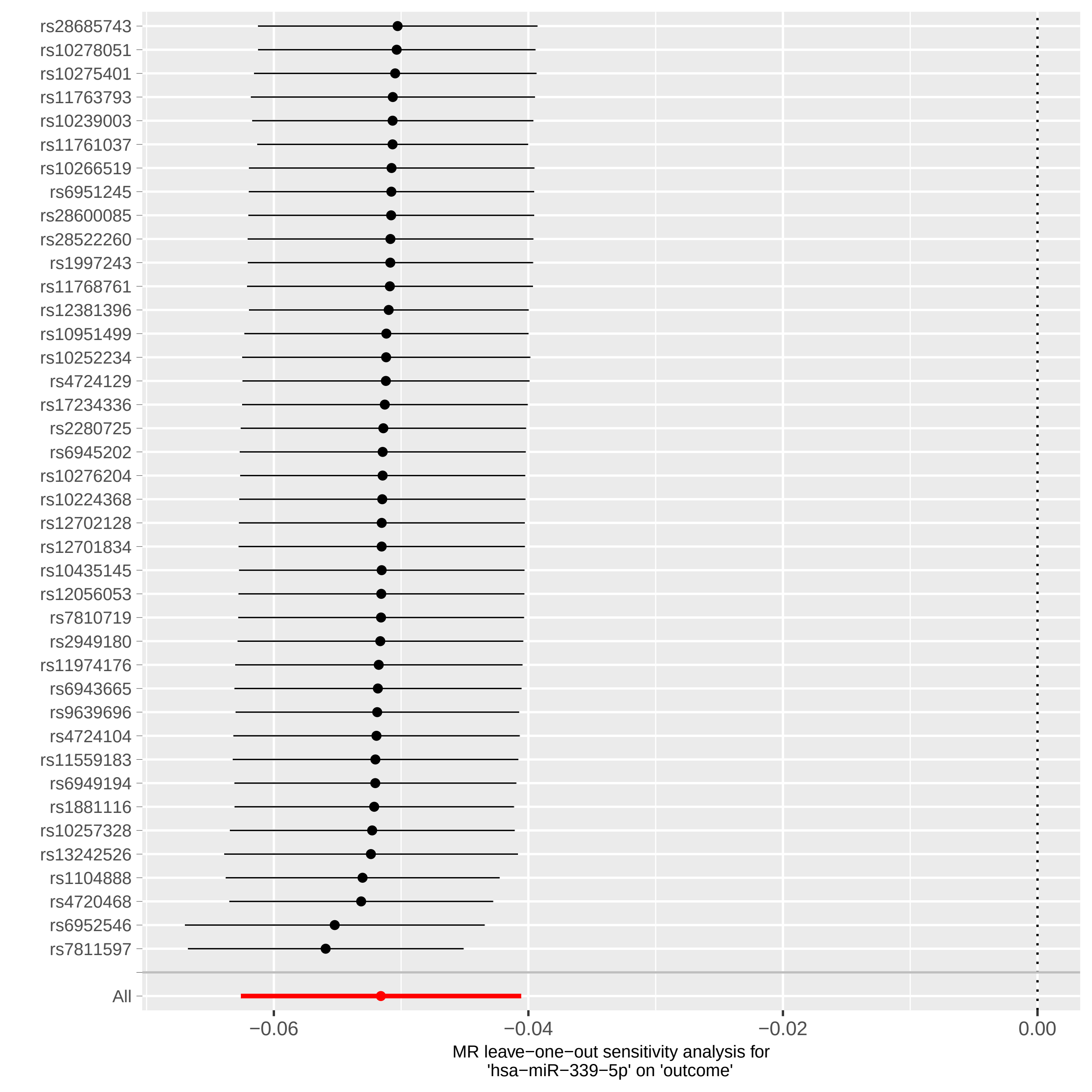
rs28685743
rs10278051
rs10275401
rs11763793
rs10239003
rs11761037
rs10266519
rs6951245
rs28600085
rs28522260
rs1997243
rs11768761
rs12381396
rs10951499
rs10252234
rs4724129
rs17234336
rs2280725
rs6945202
rs10276204
rs10224368
rs12702128
rs12701834
rs10435145
rs12056053
rs7810719
rs2949180
rs11974176
rs6943665
rs9639696
rs4724104
rs11559183
rs6949194
rs1881116
rs10257328
rs13242526
rs1104888
rs4720468
rs6952546
rs7811597
All
−0.06
−0.04
−0.02
0.00
MR leave−one−out sensitivity analysis for
'hsa−miR−339−5p' on 'outcome'
