# Supplementary material for: Identifying Putative Causal Links between MicroRNAs and Severe COVID-19 Using Mendelian Randomization
Source: Cells. 2021 Dec 11;10(12):3504. doi: 10.3390/cells10123504 (PMC8700362; doi:10.3390/cells10123504)
Supplement: Supplementary file 1 [file cells-10-03504-s001.zip › Supplementary Figures.zip!/B2_LOO/hsa-miR-550a-3pB2.pdf]

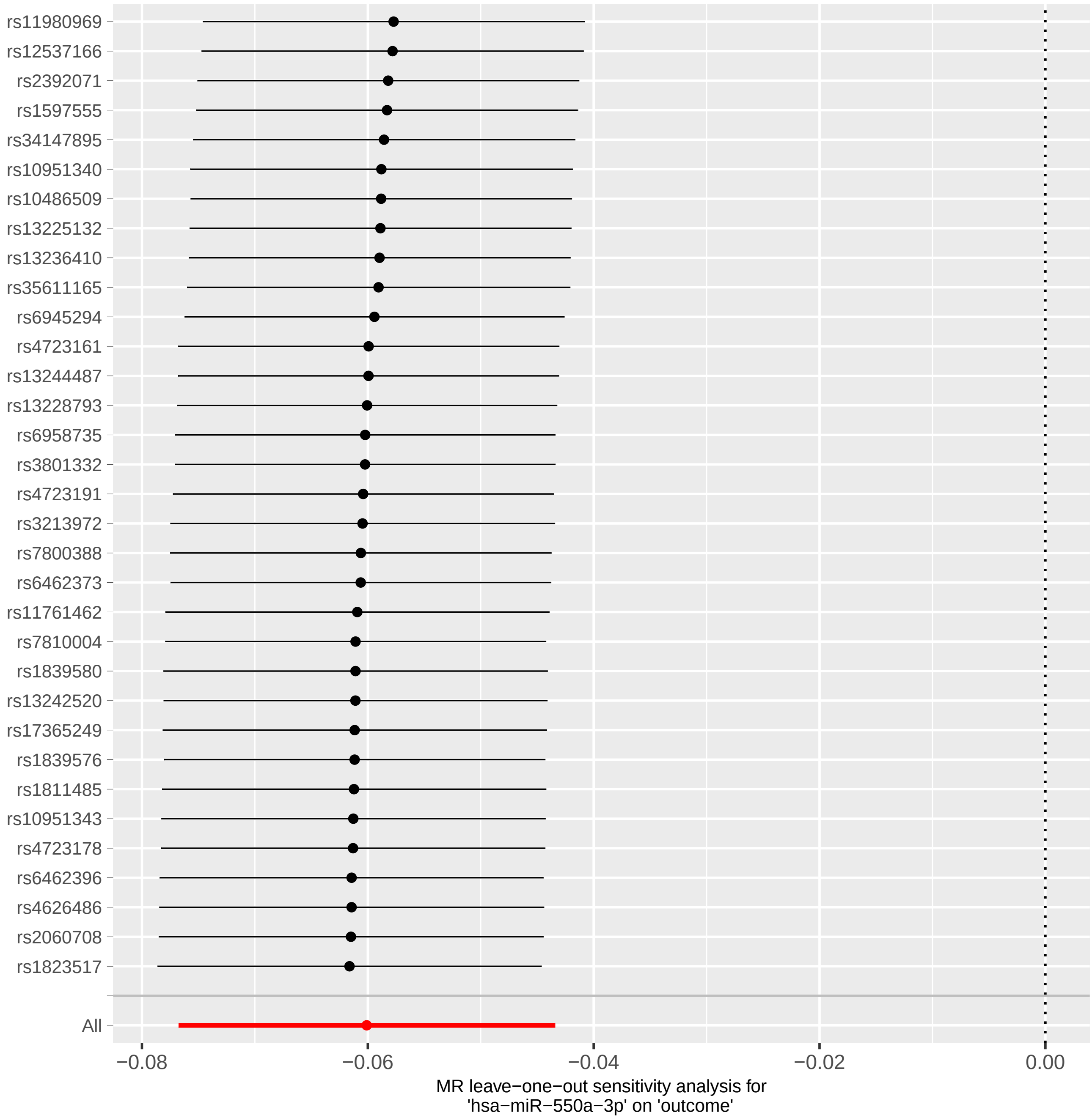

rs11980969
rs12537166
rs2392071
rs1597555
rs34147895
rs10951340
rs10486509
rs13225132
rs13236410
rs35611165
rs6945294
rs4723161
rs13244487
rs13228793
rs6958735
rs3801332
rs4723191
rs3213972
rs7800388
rs6462373
rs11761462
rs7810004
rs1839580
rs13242520
rs17365249
rs1839576
rs1811485
rs10951343
rs4723178
rs6462396
rs4626486
rs2060708
rs1823517
All
−0.08
−0.06
−0.04
−0.02
0.00
MR leave−one−out sensitivity analysis for
'hsa−miR−550a−3p' on 'outcome'
